# Prognostic Value of Parenteral Nutrition Duration on Risk of Retinopathy of Prematurity: Development and Validation of the Revised DIGIROP Clinical Decision Support Tool

**DOI:** 10.1001/jamaophthalmol.2023.2336

**Published:** 2023-06-29

**Authors:** Aldina Pivodic, Gerd Holmström, Lois E. H. Smith, Anna-Lena Hård, Chatarina Löfqvist, Abbas Al-Hawasi, Eva Larsson, Pia Lundgren, Lotta Gränse, Kristina Tornqvist, Agneta Wallin, Helena Johansson, Kerstin Albertsson-Wikland, Staffan Nilsson, Ann Hellström

**Affiliations:** 1 Department of Clinical Neuroscience, Institute of Neuroscience and Physiology, Sahlgrenska Academy, University of Gothenburg, Gothenburg, Sweden; 2 Department of Surgical Sciences/Ophthalmology, Uppsala University, Uppsala, Sweden; 3 Department of Ophthalmology, Boston Children’s Hospital, Harvard Medical School, Boston, Massachusetts; 4 Institute of Health Care Science, Sahlgrenska Academy, University of Gothenburg, Gothenburg, Sweden; 5 Division of Ophthalmology, Department of Biomedical and Clinical Sciences, Faculty of Medicine, Linköping University, Linköping, Sweden; 6 Department of Clinical Sciences, Ophthalmology, Skane University Hospital, Lund University, Lund, Sweden; 7 St Erik Eye Hospital, Stockholm, Sweden; 8 Mary MacKillop Institute for Health Research, Australian Catholic University, Melbourne, Australia; 9 Sahlgrenska Osteoporosis Centre, Institute of Medicine, University of Gothenburg, Gothenburg, Sweden; 10 Department of Physiology/Endocrinology, Institute of Neuroscience and Physiology, Sahlgrenska Academy, University of Gothenburg, Gothenburg, Sweden; 11 Institute of Biomedicine, Sahlgrenska Academy, University of Gothenburg, Gothenburg, Sweden

## Abstract

**Importance:**

The prognostic impact of parenteral nutrition duration (PND) on retinopathy of prematurity (ROP) is not well studied. Safe prediction models can help optimize ROP screening by effectively discriminating high-risk from low-risk infants.

**Objective:**

To evaluate the prognostic value of PND on ROP; to update and validate the Digital ROP (DIGIROP) 2.0 birth into prescreen and screen prediction models to include all ROP-screened infants regardless of gestational age (GA) and incorporate PND; and to compare the DIGIROP model with the Weight, IGF-1, Neonatal, and ROP (WINROP) and Postnatal Growth and ROP (G-ROP) models.

**Design, Setting, and Participants:**

This retrospective study included 11 139 prematurely born infants from 2007 to 2020 from the Swedish National Registry for ROP. Extended Poisson and logistic models were applied. Data were analyzed from August 2022 to February 2023.

**Main Outcomes and Measures:**

Any ROP and ROP requiring treatment were studied in relation to PND. ROP treatment was the outcome in DIGIROP models. Sensitivity, specificity, area under the receiver operating characteristic curve, and adjusted OR (aOR) with 95% CI were the main measures. Internal and external validations were performed.

**Results:**

Of 11 139 screened infants, 5071 (45.5%) were girls, and the mean (SD) gestational age was 28.5 (2.4) weeks. ROP developed in 3179 infants (29%), treatment was given in 599 (5%), 7228 (65%) had PND less than 14 days, 2308 (21%) had PND for 14 days or more, and 1603 (14%) had unknown PND. PND was significantly correlated with ROP severity (Spearman *r* = 0.45; *P* < .001). Infants with 14 days or more of PND vs less than 14 days had faster progression from any ROP to ROP treatment (adjusted mean difference, −0.9 weeks; 95% CI, −1.5 to −0.3; *P* = .004). Infants with PND for 14 days or more vs less than 14 days had higher odds of any ROP (aOR, 1.84; 95% CI, 1.62-2.10; *P* < .001) and of severe ROP requiring treatment (aOR, 2.20; 95% CI, 1.73-2.80; *P* < .001). Among all 11 139 infants, the DIGIROP 2.0 models had 100% sensitivity (95% CI, 99.4-100). The specificity was 46.6% (95% CI, 45.6-47.5) for the prescreen model and 76.9% (95% CI, 76.1-77.7) for the screen model. G-ROP as well as the DIGIROP 2.0 prescreen and screen models showed 100% sensitivity on a validation subset (G-ROP: sensitivity, 100%; 95% CI, 93-100; DIGIROP prescreen: sensitivity, 100%; 95% CI, 93-100; DIGIROP screen: sensitivity, 100%; 95% CI, 93-100), whereas WINROP showed 89% sensitivity (95% CI, 77-96). Specificity for each prediction model was 29% (95% CI, 22-36) for G-ROP, 38% (95% CI, 32-46) for DIGIROP prescreen, 53% (95% CI, 46-60) for DIGIROP screen at 10 weeks, and 46% (95% CI, 39-53) for WINROP.

**Conclusion and Relevance:**

Based on more than 11 000 ROP-screened infants born in Sweden, PND of 14 days or more corresponded to a significantly higher risk of having any ROP and receiving ROP treatment. These findings provide evidence to support consideration of using the updated DIGIROP 2.0 models instead of the WINROP or G-ROP models in the management of ROP.

## Key Points

**Question:**

Does parenteral nutrition duration improve the sensitivity and maintain high specificity of DIGIROP models in predicting retinopathy of prematurity (ROP) treatment?

**Findings:**

In this prognostic study, among 11 139 ROP-screened Swedish infants with 14 or more vs less than 14 days of parenteral nutrition, 64.0% vs 18.5%, respectively, had any ROP and 18.1% vs 1.6% had ROP treatment. DIGIROP 2.0 models were updated to include all ROP-screened infants regardless of gestational age and presented a 100% sensitivity, high specificity, and superiority to WINROP and G-ROP models.

**Meaning:**

The validated DIGIROP 2.0 decision support tool is suggested to be an efficient individual prediction tool for safe release of infants from unnecessary ROP screening examinations.

## Teaser

This prognostic study evaluates the prognostic value of parenteral nutrition duration on retinopathy of prematurity; updates and validates the DIGIROP 2.0 prescreen and screen prediction models to include all ROP-screened infants regardless of gestational age and incorporate parenteral nutrition duration; and compares the DIGIROP model with the WINROP and G-ROP models.

## Introduction

Retinopathy of prematurity (ROP) is a multifactorial eye disease and a major cause of visual impairment in children.^1^ Worldwide, ROP screening examinations detect and monitor the disease until it regresses or progresses to severe ROP needing treatment.^2^

The most prominent risk factors for ROP are low gestational age (GA), low birth weight (BW), low early serum insulinlike growth factor-1 (IGF-1), poor early weight gain, fluctuating oxygen concentrations, infections, and comorbidities.^3^ More parenteral nutrition and less human milk have also been identified as risk factors.^4^ Enteral nutrition, particularly with mother’s milk shortly after birth, promotes intestinal development and stimulates the cultivation of a healthier gut microbiome that is associated with lower risk of ROP.^5,6^ Likewise, early attainment of full enteral nutrition is related to lower ROP risk.^7,8,9^ Although life-saving for many infants, longer exposure to and higher volume of parenteral nutrition increase the risk of infections and reduce nutrient absorption in the premature baby.^10^

Developed in Sweden, the Weight, IGF-1, Neonatal, and ROP (WINROP) model was, to our knowledge, the first ROP prediction model proposed to identify high-risk and low-risk infants.^11,12^ It was simplified to include only GA, sex, and weekly weight gain. The Postnatal Growth and ROP (G-ROP) model, developed on approximately 7500 infants from the US and Canada, includes GA, BW, hydrocephalus, and weight gain for days 10 to 19, 20 to 29, and 30 to 39.^13,14^ Further, we developed and validated 2 prediction models for ROP treatment based on approximately 7000 Swedish infants born at 24 to 30 weeks’ GA.^15,16,17,18,19^ The Digital ROP (DIGIROP) birth model includes GA, sex, and standardized BW. Additionally, the timing for the first ROP diagnosis is included in DIGIROP screen model. Both models were developed to require 100% sensitivity. The DIGIROP birth model showed specificity of approximately 50% and the DIGIROP screen model up to approximately 80% during screening. In a contemporary Swedish cohort, approximately 50% specificity at birth was maintained, but 4 infants with severe comorbidities of 57 with ROP treatment were identified as not needing ROP screening.^19^ Inclusion of a clinical variable representing infants’ comorbidity was warranted.

Therefore, we evaluated the prognostic value of parenteral nutrition duration (PND) on ROP in this study. Furthermore, DIGIROP prediction models for ROP treatment and their clinical decision support tool were updated to include all ROP-screened infants regardless of GA and to incorporate an early cutoff for PND as well as to perform internal and external validation. Additionally, the DIGIROP outcomes were compared with those of WINROP and G-ROP.

## Methods

### Ethics

The Swedish Ethical Review Authority approved this study (Dnr 2019-02321; amendment for study extension 2007-2025 Dnr 2022-02656-02). Ethical approval was available for data extraction from the Swedish National Registry for ROP (SWEDROP) until December 31, 2025 (Dnr 2021-05134, based on Dnr 2010-117 and Dnr 2010-117/2). Parents/guardians were given the opportunity to opt out of the registry after having received the information about SWEDROP orally and in writing. This study followed the Transparent Reporting of a Multivariable Prediction Model for Individual Prognosis or Diagnosis (TRIPOD) reporting guideline and the Prediction Model Study Risk of Bias Assessment Tool (PROBAST) instrument.^20,21,22^

### Study Population

The study population included infants born from 2007 to 2020 and registered in SWEDROP (N = 11 178).^23^ SWEDROP collects information from prematurely born infants examined for ROP either routinely (ie, initially with a GA of less than 32 weeks, from 2012 with a GA of less than 31 weeks, and from 2020 with a GA of less than 30 weeks) or by indication.^24,25^ Using unique personal identification numbers, SWEDROP was linked to Swedish Neonatal Quality Register to obtain PND.^26^ Any mismatch of infants’ GA between the 2 registers as well as any other missing or questioned data were checked in the medical records.

**Figure 1.**
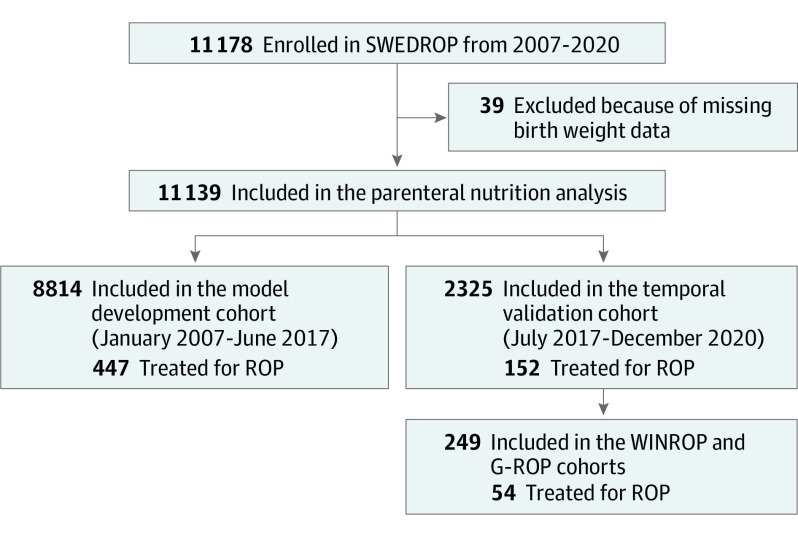
Study Flowchart G-ROP indicates Postnatal Growth and ROP; ROP, retinopathy of prematurity; SWEDROP, Swedish National Registry for ROP; WINROP, Weight, IGF-1, Neonatal, and ROP.

A total of 39 infants (0.3%) with missing BW data were excluded. In total, 11 139 infants were included (Figure 1).

#### Model Development Cohort for DIGIROP 2.0

The model development cohort included 8814 infants born from January 1, 2007, to June 30, 2017.

#### Temporal Validation Cohort for DIGIROP 2.0

The temporal validation cohort included 2325 infants born from July 1, 2017, to December 31, 2020.

#### WINROP and G-ROP Validation Cohort

For 249 infants born from July 1, 2017, to December 31, 2020, who were routinely screened and/or treated at the Queen Silvia Children’s Hospital in Gothenburg, Sweden, weekly weights were obtained from medical records to validate WINROP and G-ROP models and compare their predictive ability with DIGIROP models.

### Study Procedures

The postnatal age (PNA), postmenstrual age, and GA (by fetal ultrasonography) were defined per the American Academy of Pediatrics policy.^27^ BW SD scores (BWSDS) were calculated in infants with a GA of 24 weeks or more using the Swedish reference of approximately 800 000 singletons born from 1990 to 1999.^28^

### Study Outcomes and Predictors

The outcomes related to PND were any ROP, defined by the International Classification of ROP, and ROP treatment, as per the Early Treatment for ROP criteria, or based on the examining ophthalmologist’s assessment.^29,30^ The outcome for prediction models was ROP treatment.

PND reflects the number of days with parenteral protein and lipid supplementation. According to national and European guidelines, parenteral nutrition is initiated as early as possible after birth and is gradually increased during the following 3 to 4 days.^31,32^ Enterally, infants in Sweden receive mother’s own milk from day 1, if available, or otherwise pasteurized donor milk, increasing to an enteral target volume of 160 to 180 mL/kg per day depending on the infant’s feeding tolerance. Healthy infants are expected to reach this target during the first 2 weeks postnatally.

Predictors used for development of the DIGIROP 2.0 prescreen model were GA, sex, BW, PND (less than 14 days, 14 days or more, or unknown), and important interactions. The DIGIROP 2.0 screen model included, similar to the original publication, the log-odds of the DIGIROP 2.0 prescreen model risk estimates (that includes PND), the age and presence or not of first detection of ROP at screening occasion, and important interactions.

### Statistical Analysis

Descriptively, continuous variables were presented as means and SDs or medians and ranges, and categorical variables were presented as counts and percentages. Between-groups Fisher exact tests were used for dichotomous variables, Mantel-Haenszel χ^2^ trend tests for ordered categorical variables, and Mann-Whitney *U* tests for continuous variables. Spearman correlation was used to study correlations between ROP severity and PND.

To identify an early cutoff of PND, receiver operating characteristic analysis was performed. The cutoffs investigated were at 7 to 28 days of PND, which were considered as meaningful for an early prediction of ROP treatment. The selected cutoff at 14 days had the highest area under the receiver operating characteristic curve (AUC) and maximized sensitivity and specificity (Youden index). The associations between PND and ROP were studied using logistic regression adjusting for GA, BW, and sex. Odds ratios (ORs), adjusted ORs (aORs), and 95% CI were calculated. In addition, risk differences were described in crude absolute terms using Meittinen-Nurminen 95% confidence limits.

The DIGIROP 2.0 prescreen model was developed including all ROP-screened infants using extended Poisson regression.^17,33,34,35^ The first model included variables from the DIGIROP 1.0 birth model, which was extended by including categorized PND and important interactions, and therefore renamed to the DIGIROP 2.0 prescreen model. The selected model had the lowest Akaike information criterion value. The parameter estimate, standard error, hazard ratio with 95% CIs, and *P* value were presented. The estimated probability for ROP treatment was calculated as 1 − survival probability. Survival probability was obtained by exp(−H[*t*]) and H(*t*) by numerical integration of the hazard function for 20 follow-up weeks.

The DIGIROP 2.0 screen model was developed including all ROP-screened infants using logistic regression models for PNA from 6 to 14 weeks. The same variables as those included in the original publication were used.^18^

The models’ predictive ability was described by sensitivity, specificity, cumulative specificity, positive predictive value, negative predictive value, accuracy, and AUC. For the DIGIROP screen model, the specificity for each week from weeks 6 to 14 was based on the number of infants discharged from ROP examinations that week or previously and was termed *cumulative specificity*. Calibration plots and Hosmer-Lemeshow test were performed to evaluate observed vs estimated probabilities. Internal validation of the models was performed using 10-fold cross-validation. External validation was performed on a temporally different Swedish cohort to evaluate the models’ transportability in time. The model’s sensitivity and specificity were compared with the WINROP model (2006 to 2009) and G-ROP model (2018 to 2020) in a subset of infants from the temporal validation cohort.^12,14^ Superiority was evaluated by first comparing sensitivity, requiring achievement of 100%. Then, the Sign test was used to demonstrate superiority of one method over the other considering specificity. To obtain weights for postnatal days 10, 19, 20, 29, 30, and 39 in the G-ROP model, linear interpolation was applied. In case of missing data, the infant was deemed to need screening for both WINROP and G-ROP.

All tests were 2-sided. The significance level was *P* < .05. No adjustment for multiple comparisons was made. Only positive associations between PND and ROP were to be demonstrated. All analyses were performed using SAS software version 9.4 (SAS Institute) and R version 4.2.0 (The R Foundation).

## Results

### Study Population

Of 11 139 infants included in the study, 5071 (45.5%) were girls, the mean (SD; range) GA was 28.5 (2.4; 21.9-39.4) weeks, the mean (SD) BW was 1172 (384) g, and the mean (SD) BWSDS was −1.11 (1.44). Any ROP was observed in 3179 infants (28.5%), and the median (range) time of first ROP diagnosis was 8.4 (0.9-24.7) weeks. There were 599 infants (5.4%) treated for ROP, with a median (range) first ROP treatment provided at 12.6 (6.3-28.3) weeks (Table 1).

**Table 1.** Infant Characteristics by Model Cohorts and by Parenteral Nutrition Duration (PND)

Characteristic	No. (%)							
	Total (N = 11 139)	Model cohort			PND			
		Development (n = 8814)^a^	Validation (n = 2325)^b^	*P* value^c^	<14 d (n = 7228)	≥14 d (n = 2308)	*P* value^c^	Unknown (n = 1603)
Gender								
Boys	6068 (54.5)	4806 (54.5)	1262 (54.3)	.83	3869 (53.5)	1270 (55.0)	.21	929 (58.0)
Girls	5071 (45.5)	4008 (45.5)	1063 (45.7)		3359 (46.5)	1038 (45.0)		674 (42.0)
Gestational age, wk								
Mean (SD)	28.5 (2.4)	28.6 (2.4)	28.0 (2.3)	<.001	29.2 (1.9)	26.2 (2.1)	<.001	28.7 (2.3)
Median (range)	28.9 (21.9-39.4)	29.0 (21.9-39.4)	28.4 (22.1-35.9)		29.4 (21.9-39.4)	26.0 (21.9-35.0)		29.1 (22.1-36.1)
<24	474 (4.3)	343 (3.9)	131 (5.6)	<.001	80 (1.1)	337 (14.6)	<.001	57 (3.6)
24-30	9196 (82.5)	7095 (80.5)	2101 (90.4)		6029 (83.4)	1931 (83.7)		1236 (77.1)
≥31	1469 (13.2)	1376 (15.6)	93 (4.0)		1119 (15.5)	40 (1.7)		310 (19.3)
Birth weight, g								
Mean (SD)	1172 (384)	1192 (389)	1096 (356)	<.001	1268 (349)	847 (303)	<.001	1207 (387)
Median (range)	1164 (307-3540)	1186 (307-3245)	1095 (340-3540)		1260 (382-3245)	786 (307-3540)		1205 (370-2695)
Birth weight SDS								
Mean (SD)	−1.11 (1.44)	−1.10 (1.43)	−1.17 (1.49)	.14	−1.08 (1.40)	−1.24 (1.61)	.02	−1.13 (1.43)
Median (range)	−0.8 (−9.1 to 5.4)	−0.8 (−9.1 to 4.9)	−0.8 (−6.9 to 5.4)		−0.8 (−8.1 to 4.9)	−0.9 (−9.1 to 5.4)		−0.8 (−8.7 to 4.0)
Missing data, No.	474	343	131		80	337		57
Any ROP	3179 (28.5)	2416 (27.4)	763 (32.8)	<.001	1340 (18.5)	1477 (64.0)	<.001	362 (22.6)
Maximum ROP stage								
No ROP	7960 (71.5)	6398 (72.6)	1562 (67.2)	<.001	5888 (81.5)	831 (36.0)	<.001	1241 (77.4)
Stage 1	956 (8.6)	715 (8.1)	241 (10.4)		559 (7.7)	290 (12.6)		107 (6.7)
Stage 2 not treated	1102 (9.9)	874 (9.9)	228 (9.8)		492 (6.8)	479 (20.8)		131 (8.2)
Stage 3 not treated	521 (4.7)	379 (4.3)	142 (6.1)		174 (2.4)	289 (12.5)		58 (3.6)
Stage 5 not treated	1 (0.0)	1 (0.0)	0		0	1 (0.0)		0
Treated ROP	599 (5.4)	447 (5.1)	152 (6.5)		115 (1.6)	418 (18.1)		66 (4.1)
PNA at first ROP diagnosis, wk								
Mean (SD)	8.6 (2.3)	8.5 (2.3)	8.6 (2.3)	.23	7.9 (2.2)	9.2 (2.2)	<.001	8.6 (2.4)
Median (range)	8.4 (0.9-24.7)	8.3 (1.3-24.7)	8.6 (0.9-17.6)		7.6 (0.9-17.6)	9.0 (4.0-24.7)		8.3 (4.1-17.3)
Missing data, No.	7960	6398	1562		5888	831		1241
PNA at first ROP treatment, wk								
Mean (SD)	13.0 (3.0)	12.9 (2.9)	13.2 (3.3)	.64	13.5 (3.1)	12.9 (3.0)	.04	12.9 (2.9)
Median (range)	12.6 (6.3-28.3)	12.4 (6.3-28.3)	12.6 (7.7-26.3)		12.9 (6.3-24.3)	12.4 (7.0-28.3)		12.5 (7.9-19.9)
Missing data, No.	10 540	8367	2173		7113	1890		1537
Time between first ROP diagnosis and treatment, wk								
Mean (SD)	3.9 (3.0)	3.9 (2.8)	3.8 (3.3)	.50	4.9 (3.1)	3.6 (2.9)	<.001^d^	3.8 (2.8)
Median (range)	3.1 (0.0-19.1)	3.3 (0.1-18.9)	3.1 (0.0-19.1)		4.3 (0.1-15.9)	3.0 (0-19.1)		3.2 (0.1-11.6)
Missing data, No.	10 540	8367	2173		7113	1890		1537
Time of parenteral nutrition, d								
Mean (SD)	10.8 (16.7)	9.9 (15.8)	13.7 (18.9)	<.001	4.4 (4.2)	30.8 (23.9)	<.001	NA
Median (range)	6 (0-279)	5 (0-279)	9 (0-257)		4 (0-13)	23 (14-279)		NA
Missing data, No.	1603	1569	34		0	0		NA

Abbreviations: NA, not applicable; PNA, postnatal age; ROP, retinopathy of prematurity; SDS, standard deviation score.

^a^ The development cohort included Swedish National Registry for ROP data collected from January 1, 2007, to June 30, 2017.

^b^ The temporal validation cohort included Swedish National Registry for ROP data collected from July 1, 2017, to December 31, 2020.

^c^ For between-group tests, Fisher exact test was used for dichotomous variables, Mantel-Haenszel χ^2^ trend test for ordered categorical variables, and Mann-Whitney *U* test for continuous variables.

^d^ Significant also after adjustment for gestational age (−0.9; 95% CI, −1.5 to −0.3; *P* = .004).

The model development cohort for DIGIROP 2.0 models included 8814 infants (79.1%), and the temporal validation cohort included 2325 (20.9%). Compared with the model development cohort, more infants in the temporal validation cohort had lower GA (mean [SD] GA, 28.0 [2.3] vs 28.6 [2.4] weeks), lower BW (mean [SD] BW, 1096 [356] vs 1192 [389] g), had any ROP (763 [32.8%] vs 2416 [27.4%]), and received ROP treatment (152 [6.5%] vs 447 [5.1%]) (Table 1).

### PND and ROP

**Figure 2.**
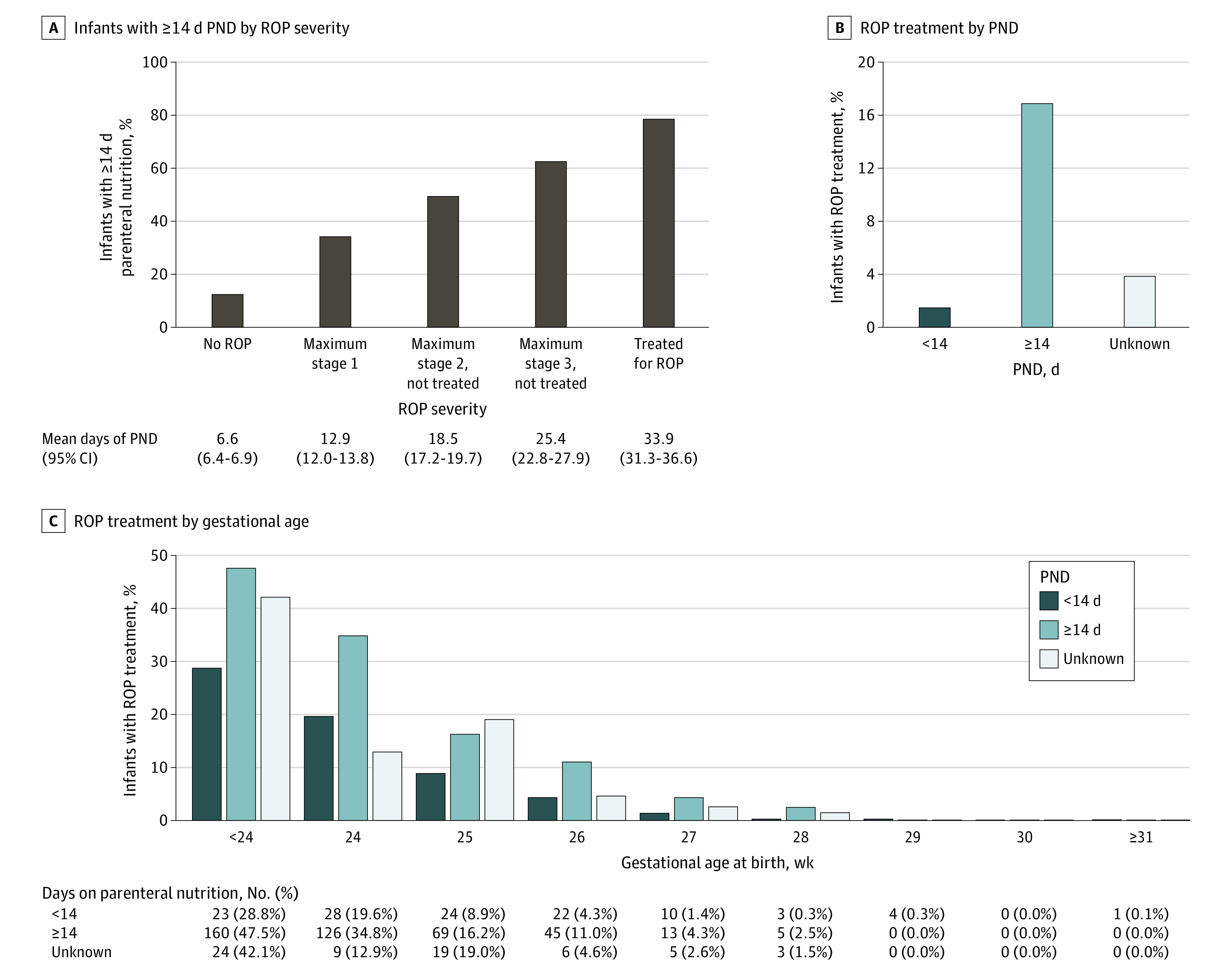
Percentage of Infants With Parenteral Nutrition Duration (PND) of 14 Days or More by Retinopathy of Prematurity (ROP) Severity and ROP Treatment by PND and Gestational Age A, Mean duration of parenteral nutrition is provided by ROP severity. Error bars indicate 95% CIs. B, Infants with ROP treatment are presented per PN duration category. C, Infants with ROP treatment are presented per PND category for different gestational ages.

Among the whole cohort, infants received a mean (SD) of 10.8 (16.7) days of PND. A total of 7228 infants (64.9%) received PND for less than 14 days, 2308 (20.7%) received PND for 14 days or more, and 1603 (14.4%) had unknown PND (Table 1). The proportion of infants with 14 days or more days of PND and number of days on parenteral nutrition increased gradually with ROP severity (Spearman *r* = 0.45; *P* < .001) (Figure 2A).

Compared with those with PND less than 14 days, infants with PND for 14 days or more had lower GA (mean [SD], 26.2 [2.1] vs 29.2 [1.9] weeks), lower BW (mean [SD], 847 [303] vs 1268 [349] g), more infants had any ROP (1477 [64.0%] vs 1340 [18.5%]), and more had treated ROP (418 [18.1%] vs 115 [1.6%]) (Table 1). Overall and GA-stratified data are presented in Figure 2B and C and eFigure 1 in Supplement 1.

Disease progression from the first detection of ROP to first ROP treatment was faster in infants receiving PND for 14 days or more compared with less than 14 days (median [range], 3.0 [0-19.1] vs 4.3 [0.1-15.9] weeks; GA-adjusted mean difference, −0.9; 95% CI, −1.5 to −0.3; *P* = .004) (Table 1).

After adjustment for GA, BW, and sex, longer PND was associated with more ROP of any stage (aOR per 1-week increase, 1.16; 95% CI, 1.13-1.20; *P* < .001) (Table 2). The AUC for PND alone was 0.77. Categorized, PND of 14 or more days vs less than 14 days showed an aOR of 1.84 (95% CI, 1.62-2.10; *P* < .001) for any ROP. The absolute risk difference was 45% (95% CI, 43-48).

**Table 2.** Unadjusted and Adjusted Logistic Regression for Any Retinopathy of Prematurity (ROP) and ROP Treatment Studying Parenteral Nutrition (PN) as the Main Association Variable

Outcome	Events, No. (%)	Unadjusted			Adjusted for GA, BW, and sex		
		OR (95% CI)	*P* value	AUC	aOR (95% CI)	*P* value	AUC
Any ROP							
Duration of PN (per 1-wk increase)	NA	1.74 (1.68-1.80)	<.001	0.77	1.16 (1.13-1.20)	<.001	0.87
Duration of PN (dichotomized), d							
<14	1340 (18.5)	1 [Reference]	NA	0.69	1 [Reference]	NA	0.87
≥14	1477 (64.0)	7.81 (7.04-8.66)	<.001		1.84 (1.62-2.10)	<.001	
Unknown	362 (22.6)	1.28 (1.12-1.46)	.002		0.87 (0.74-1.02)	.08	
ROP treatment							
Duration of PN (per 1-wk increase)	NA	1.36 (1.32-1.39)	<.001	0.83	1.12 (1.09-1.15)	<.001	0.93
Duration of PN (dichotomized), d							
<14	115 (1.6)	1 [Reference]	NA	0.78	1 [Reference]	NA	0.93
≥14	418 (18.1)	13.68 (11.06-16.92)	<.001		2.20 (1.73-2.80)	<.001	
Unknown	66 (4.1)	2.66 (1.95-3.61)	<.001		1.40 (1.00-1.97)	.05	

Abbreviations: aOR, adjusted odds ratio; AUC, area under the receiver operating characteristic curve; BW, birth weight; GA, gestational age; NA, not applicable; OR, odds ratio.

PND was statistically significantly associated with ROP treatment (aOR per 1-week increase, 1.12; 95% CI, 1.09-1.15; *P* < .001) (Table 2). The AUC for PND alone was 0.83. Infants with PND for 14 days or more had significantly more ROP treatment than those with less than 14 days (aOR, 2.20; 95% CI, 1.73-2.80; *P* < .001). The absolute risk difference was 17% (95% CI, 15-18). Similar conclusions were drawn for the model development and temporal validation cohorts (eTables 1 and 2 in Supplement 1).

### Update of the DIGIROP 2.0 Prescreen Prediction Model for ROP Treatment Including PND

**Figure 3.**
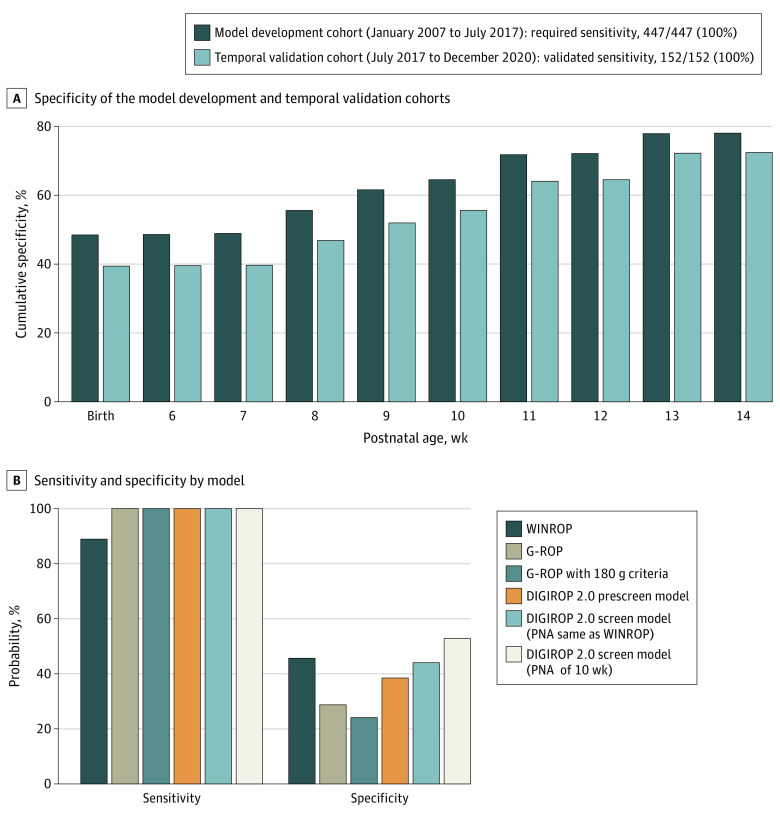
Sensitivity and Specificity for the DIGIROP 2.0 Prescreen and Screen Models Including Parenteral Nutrition Duration (Cumulatively Over Postnatal Age [PNA] Models) and for WINROP, G-ROP, and the DIGIROP 2.0 Prescreen and Screen Models on a Subset of Temporal Validation Cohort A, Cumulative specificity in the model development and temporal validation cohorts. B, Sensitivity and specificity of the WINROP, G-ROP, and DIGIROP 2.0 models. WINROP uses the algorithm from Hellström et al,^12^ G-ROP uses the algorithm from Binenbaum et al,^13^ and G-ROP with the 180 g criteria uses the algorithm from Binenbaum et al.^14^ The DIGIROP 2.0 screen model uses longitudinal screening data with equally long time, as longitudinal weights are used by WINROP. DIGIROP was superior to WINROP based on sensitivity. Considering the specificity, DIGIROP models were superior to G-ROP models. DIGIROP indicates Digital ROP; G-ROP, Postnatal Growth and ROP; WINROP, Weight, IGF-1, Neonatal, and ROP.

In the DIGIROP 2.0 prescreen model, BWSDS was replaced by BW, including all ROP-screened infants. Categorized PND (less than 14 days, 14 days or more, and unknown) was added to capture high-risk infants who cannot be captured given only GA, BW, and sex. The interaction between sex and PND was significant (eFigure 2 in Supplement 1), showing more or similar ROP treatment received by girls compared with boys among infants who received 14 days or more of PND, as opposed to those who received less than 14 days of PND, where girls needed ROP treatment less than boys. The final model is presented in eTable 3 in Supplement 1 and the estimated probabilities in eFigure 3 in Supplement 1. The model was well calibrated (Hosmer-Lemeshow test, *P* = .57) (eTable 3 in Supplement 1), and the calibration plot of observed vs estimated probabilities well distributed around the diagonal (eFigure 4 in Supplement 1). The AUC was 0.93. Given the required 100% (95% CI, 99.2-100) sensitivity, the specificity was 48.5% (95% CI, 47.4-49.5) (Figure 3). The percentage of infants discharged from screening by GA is given in eFigure 5A in Supplement 1.

### Internal and External Validation of the DIGIROP 2.0 Prescreen Prediction Model for ROP Treatment Including PND

Internal validation using cross-validation showed a specificity of 47.4% (eFigure 6 in Supplement 1). The obtained sensitivity on the temporally different Swedish validation cohort was 100% (95% CI, 97.6-100) and the specificity was 39.4% (95% CI, 37.3-41.5) (Figure 3A). The lower specificity in the validation cohort was secondary to lower GA due to increased survival of more immature infants and fewer infants with a GA of 30 weeks (93 of 2325 [4.0%] vs 1376 of 8814 [15.6%]) (Table 1). Considering the total population, the specificity was 46.6% (95% CI, 45.6-47.5).

In the temporal validation cohort, 118 of 337 infants (35.0%) born at 28 weeks’ GA could be discharged, 288 of 463 (62.2%) born at 29 weeks’ GA could be discharged, 374 of 420 (89.0%) born at 30 weeks’ GA could be discharged, and 52 of 93 (55.9%) born at 31 weeks’ GA or later (compared with 1116 of 1376 [81.1%] in the model development cohort born at 31 weeks’ GA or later) (eFigure 5 in Supplement 1). No infants born at 24 weeks’ GA or less were discharged.

### Update of the DIGIROP 2.0 Screen Prediction Model for ROP Treatment Including PND in the Risk Estimates (Log-Odds) From DIGIROP 2.0 Prescreen Model

The final logistic models for the DIGIROP 2.0 screen model, one per each PNA week 6 to 14, are presented in eTable 4 in Supplement 1. The AUC ranged between 0.93 and 0.95. Hosmer-Lemeshow test (eTable 4 in Supplement 1) and calibration plot (eFigure 7A in Supplement 1) reported well-calibrated models. The GA-specific cutoffs are presented in eTable 5 in Supplement 1. For the required 100% sensitivity, the specificity increased from 44.9% (95% CI, 43.8-46.0) to 75.9% (95% CI, 75.0-76.9) and cumulative specificity from 48.6% (95% CI, 47.5-49.7) to 78.0% (95% CI, 77.1-78.9) for PNA 6 to 14 weeks (eTable 6 and eFigure 8 in Supplement 1; Figure 3A).

### Internal and External Validation of the DIGIROP 2.0 Screen Prediction Model for ROP Treatment Including PND in the Risk Estimates (Log-Odds) From the DIGIROP 2.0 Prescreen Model

Internal validation using cross-validation showed similar results to those obtained in the main analysis (eFigure 6 in Supplement 1). The models were well-calibrated on the external validation cohort (eFigure 7B in Supplement 1). The sensitivity was 100% for all PNA weeks (eTable 6 in Supplement 1). The specificity ranged between 35.1% (95% CI, 33.1-37.1) and 69.8% (95% CI, 67.8-71.7), and cumulative specificity between 39.5% (95% CI, 37.5-41.6) and 72.4% (95% CI, 70.5-74.3) (eFigure 8 in Supplement 1; Figure 3A). Considering the total population, the cumulative specificity increased from 46.6% (95% CI, 45.6-47.5) to 76.9% (95% CI, 76.1-77.7). Bar graphs representing the percentage of infants discharged from screening by GA are shown in eFigure 5 in Supplement 1.

### DIGIROP 2.0 Clinical Decision Support Tool Including PND Compared With WINROP and G-ROP 

Validation of WINROP and G-ROP was performed including 249 infants, including 54 (21.7%) needing ROP treatment. Infants with missing weight (WINROP, 33 of 249 [13.3%]; G-ROP, 23 of 249 [9.2%]) were deemed needing screening. The DIGIROP models and G-ROP criteria were superior to WINROP considering sensitivity, and for specificity, DIGIROP models were superior to G-ROP criteria (Figure 3B).

## Discussion

Including all Swedish ROP-screened infants regardless of GA in the SWEDROP from 2007 to 2020, we showed a strong prognostic value of days of parenteral nutrition on any ROP and ROP needing treatment. After adjustment, infants with 14 days or more of PND had 84% higher odds of any ROP and 120% higher odds of ROP treatment than those with less than 14 days PND. Including GA, BW, sex, PND, and status and age at the first ROP diagnosis, DIGIROP 2.0 prediction models were updated and validated into a safe (sensitivity, 100%; 95% CI, 99-100) clinical decision support tool with a specificity of 47% (95% CI, 46-48) to 77% (95% CI, 76-78) that could be applied by physicians using the online application.^15^

Vanhaesebrouck and colleagues^36^ showed in 2008 among 412 infants (26% ROP treatment) that PND, GA, BW, and length of oxygen were independent predictors for any ROP. Niwald et al^37^ showed that PND for more than 10 days was a predictor for ROP needing treatment among 118 infants. Petrachkova et al^38^ developed a prognostic model for type 1 ROP (69 infants; 42% with type 1 ROP), with PND for more than 13 days being one of the significant predictors. Interestingly, the same cutoff for PND was selected as that in our cohort including more than 11 000 infants. We investigated days 7 to 28 to enable early prognosis; a 14-day cutoff had the highest predictive ability. Related to this, Porcelli and Weaver Jr^4^ found that volume of parenteral nutrition during week 2, but not week 1, was related to severe ROP outcome.

PND was greater in girls than in boys, especially for lower GA, where ROP needing treatment is more frequent. Infants with longer PND had faster progression of the disease independently of GA. Further investigation of the mechanism behind early sex-specific effects of parenteral nutrition and its relation to intestinal and neurovascular development as well as accelerated progression of severe ROP is needed.

An unbiased way of comparing different ROP models is through application on the same data. In our validation subset, WINROP showed higher specificity (46%; 95% CI, 39-53) than G-ROP (29%; 95% CI, 22-36), G-ROP with the 180 g criteria (24%; 95% CI, 18-31), and the DIGIROP prescreen model (38%; 95% CI, 32-46). However, 6 of 54 infants needing ROP treatment were missed. G-ROP and DIGIROP models showed 100% sensitivity (95% CI, 93-100). Although not required, WINROP allows usage of weekly weights until ROP treatment or 40 weeks’ postmenstrual age, while G-ROP and the DIGIROP prescreen model provide an earlier estimation up to 39 days’ and 14 days’ PNA, respectively.

### Strengths and Limitations

This study’s strength is that it comprises the ROP-screened Swedish population from 2007 to 2020, excluding only 39 of 11 178 infants with missing data. However, this study has limitations. Register data may include potential editing errors. Additionally, due to the Swedish population being homogenous in terms of ethnicity, neonatal care, and socioeconomic status, the DIGIROP models need to be thoroughly validated on other populations and settings before being implemented.

## Conclusions

Our study demonstrated a substantial prognostic value of PND on any ROP and ROP requiring treatment. Infants with PND of 14 days or more were at significantly higher risk of needing treatment. Continuous research on neonatal nutrition for premature babies is warranted.

Further, we updated and externally validated DIGIROP 2.0 prediction models and their clinical decision support tool to achieve 100% sensitivity and high specificity. Considering both sensitivity and specificity, the DIGIROP clinical decision support tool was shown to be superior to WINROP and G-ROP.

## Notes

Supplement 1. **eTable 1.** Infant Characteristics for the Model Development Cohort and Temporal Validation Cohort by Parenteral Nutrition Duration **eTable 2.** Unadjusted and Adjusted Logistic Regression for Any Retinopathy of Prematurity (ROP) and ROP Treatment Studying Parenteral Nutrition as the Main Association Variable for the Model Development Cohort and Temporal Validation Cohort **eTable 3.** DIGIROP Pre-Screen 2.0 Prediction Model for Retinopathy of Prematurity Treatment Including Parenteral Nutrition Duration **eTable 4.** DIGIROP-Screen 2.0 Prediction Models for Retinopathy of Prematurity Treatment Including Parenteral Nutrition Duration **eTable 5.** Cutoffs for DIGIROP Pre-Screen 2.0 and DIGIROP Screen 2.0 Including Parenteral Nutrition Duration **eTable 6.** Sensitivity, Specificity, Cumulative Specificity, PPV, NPV, Model Accuracy, and 95% CI for DIGIROP Pre-Screen 2.0 and DIGIROP Screen 2.0 Including Parenteral Nutrition Duration for the Model Development and Temporal Validation Cohort **eFigure 1.** Bar Graphs for Percentage Infants With Any ROP by PN Duration, Gestational Age, and Overall **eFigure 2.** Bar Graphs for Percentage Infants with ROP Treatment by PN Duration, Gestational Age, and Sex **eFigure 3.** Estimated DIGIROP Pre-Screen 2.0 Probability for ROP Treatment Including Parenteral Nutrition Duration by Gestational Age **eFigure 4.** Calibration Plot for DIGIROP Pre-Screen 2.0 Including Parenteral Nutrition Duration for Model Development Cohort and Temporal Validation Cohort **eFigure 5.** Infants Discharged From Screening by PNA and GA **eFigure 6.** Internal Validation of DIGIROP Pre-Screen 2.0 and DIGIROP Screen 2.0 Including Parenteral Nutrition Duration Using 10-Fold Cross-Validation **eFigure 7.** Calibration Plot for DIGIROP-Screen 2.0 Including Parenteral Nutrition Duration for (A) Model Development Cohort and (B) Temporal Validation Cohort **eFigure 8.** Sensitivity and Specificity for DIGIROP Pre-Screen 2.0 and DIGIROP Screen 2.0 Including Parenteral Nutrition Duration

Supplement 2. Data Sharing Statement

## References

[1] Blencowe H, Lawn JE, Vazquez T, Fielder A, Gilbert C. Preterm-associated visual impairment and estimates of retinopathy of prematurity at regional and global levels for 2010. Pediatr Res. 2013;74(suppl 1):35-49. doi:10.1038/pr.2013.205 24366462 PMC3873709

[2] Mora JS, Waite C, Gilbert CE, Breidenstein B, Sloper JJ. A worldwide survey of retinopathy of prematurity screening. Br J Ophthalmol. 2018;102(1):9-13. doi:10.1136/bjophthalmol-2017-310709 28855196

[3] Hellström A, Smith LE, Dammann O. Retinopathy of prematurity. Lancet. 2013;382(9902):1445-1457. doi:10.1016/S0140-6736(13)60178-6 23782686 PMC4389630

[4] Porcelli PJ, Weaver RG Jr. The influence of early postnatal nutrition on retinopathy of prematurity in extremely low birth weight infants. Early Hum Dev. 2010;86(6):391-396. doi:10.1016/j.earlhumdev.2010.05.015 20561759

[5] Mammas IN, Spandidos DA. Retinopathy of prematurity and neonatal gut microbiome: an interview with Professor Dimitra Skondra, Associate Professor of Ophthalmology and Vitreoretinal Surgeon at The University of Chicago (USA). Exp Ther Med. 2020;20(6):294. doi:10.3892/etm.2020.9424 33209138 PMC7668155

[6] Skondra D, Rodriguez SH, Sharma A, Gilbert J, Andrews B, Claud EC. The early gut microbiome could protect against severe retinopathy of prematurity. J AAPOS. 2020;24(4):236-238. doi:10.1016/j.jaapos.2020.03.010 32707176 PMC7680397

[7] Sanghvi KP, Joshi P, Nabi F, Kabra N. Feasibility of exclusive enteral feeds from birth in VLBW infants >1200 g–an RCT. Acta Paediatr. 2013;102(7):e299-e304. doi:10.1111/apa.12254 23621289

[8] Jajoo M, Singh A, Arora N, Bhaskar V, Mandal A. Early total versus gradually advanced enteral nutrition in stable very-low-birth-weight preterm neonates: a randomized, controlled trial. Indian J Pediatr. 2022;89(1):25-30. doi:10.1007/s12098-021-03778-6 34117622

[9] Nangia S, Vadivel V, Thukral A, Saili A. Early total enteral feeding versus conventional enteral feeding in stable very-low-birth-weight infants: a randomised controlled trial. Neonatology. 2019;115(3):256-262. doi:10.1159/000496015 30699425

[10] Patel AL, Taylor SN. Dilemmas in initiation of very preterm infant enteral feeds—when, what, how? J Perinatol. 2023;43(1):108-113. doi:10.1038/s41372-022-01564-6 36447040

[11] Löfqvist C, Hansen-Pupp I, Andersson E, . Validation of a new retinopathy of prematurity screening method monitoring longitudinal postnatal weight and insulinlike growth factor I. Arch Ophthalmol. 2009;127(5):622-627. doi:10.1001/archophthalmol.2009.69 19433710

[12] Hellström A, Hård AL, Engström E, . Early weight gain predicts retinopathy in preterm infants: new, simple, efficient approach to screening. Pediatrics. 2009;123(4):e638-e645. doi:10.1542/peds.2008-2697 19289449

[13] Binenbaum G, Bell EF, Donohue P, ; G-ROP Study Group. Development of modified screening criteria for retinopathy of prematurity: primary results from the Postnatal Growth and Retinopathy of Prematurity study. JAMA Ophthalmol. 2018;136(9):1034-1040. doi:10.1001/jamaophthalmol.2018.2753 30003216 PMC6142979

[14] Binenbaum G, Tomlinson LA, de Alba Campomanes AG, ; Postnatal Growth and Retinopathy of Prematurity (G-ROP) Study Group. Validation of the postnatal growth and retinopathy of prematurity screening criteria. JAMA Ophthalmol. 2020;138(1):31-37. doi:10.1001/jamaophthalmol.2019.4517 31725856 PMC6865315

[15] DIGIROP. Homepage. Accessed April 11, 2023. http://www.digirop.com/index.html

[16] University of Gothenburg. The Sahlgrenska Center for Pediatric Ophthalmology Research. Accessed April 11, 2023. https://www.gu.se/en/research/pediatric-ophthalmology

[17] Pivodic A, Hård AL, Löfqvist C, . Individual risk prediction for sight-threatening retinopathy of prematurity using birth characteristics. JAMA Ophthalmol. 2020;138(1):21-29. doi:10.1001/jamaophthalmol.2019.4502 31697330 PMC6865304

[18] Pivodic A, Johansson H, Smith LEH, . Development and validation of a new clinical decision support tool to optimize screening for retinopathy of prematurity. Br J Ophthalmol. 2022;106(11):1573-1580. doi:10.1136/bjophthalmol-2020-318719 33980506 PMC8627649

[19] Pivodic A, E H Smith L, Hård AL, . Validation of DIGIROP models and decision support tool for prediction of treatment for retinopathy of prematurity on a contemporary Swedish cohort. Br J Ophthalmol. Published online March 11, 2022. doi:10.1136/bjophthalmol-2021-320738 35277395 PMC10359565

[20] Collins GS, Reitsma JB, Altman DG, Moons KG. Transparent reporting of a multivariable prediction model for individual prognosis or diagnosis (TRIPOD): the TRIPOD statement. BMC Med. 2015;13:1. doi:10.1186/s12916-014-0241-z 25563062 PMC4284921

[21] Moons KGM, Wolff RF, Riley RD, . PROBAST: a tool to assess risk of bias and applicability of prediction model studies: explanation and elaboration. Ann Intern Med. 2019;170(1):W1-W33. doi:10.7326/M18-1377 30596876

[22] Wolff RF, Moons KGM, Riley RD, ; PROBAST Group†. PROBAST: a tool to assess the risk of bias and applicability of prediction model studies. Ann Intern Med. 2019;170(1):51-58. doi:10.7326/M18-1376 30596875

[23] Swedish National Registry for Retinopathy of Prematurity. Välkommen till SWEDROP. Accessed April 11, 2023. https://www.medscinet.com/ROP/

[24] Holmström GE, Hellström A, Jakobsson PG, Lundgren P, Tornqvist K, Wallin A. Swedish National Register for Retinopathy of Prematurity (SWEDROP) and the evaluation of screening in Sweden. Arch Ophthalmol. 2012;130(11):1418-1424. doi:10.1001/archophthalmol.2012.2357 23143441

[25] Holmström G, Hellström A, Gränse L, . New modifications of Swedish ROP guidelines based on 10-year data from the SWEDROP register. Br J Ophthalmol. 2020;104(7):943-949. doi:10.1136/bjophthalmol-2019-314874 31676594

[26] Swedish Neonatal Quality Register. Välkommen till Neonatalvårdsregistret. Accessed April 11, 2023. https://www.medscinet.com/pnq/default.aspx

[27] Engle WA; American Academy of Pediatrics Committee on Fetus and Newborn. Age terminology during the perinatal period. Pediatrics. 2004;114(5):1362-1364. doi:10.1542/peds.2004-1915 15520122

[28] Niklasson A, Albertsson-Wikland K. Continuous growth reference from 24th week of gestation to 24 months by gender. BMC Pediatr. 2008;8:8. doi:10.1186/1471-2431-8-8 18307822 PMC2294116

[29] International Committee for the Classification of Retinopathy of Prematurity. The International Classification of Retinopathy of Prematurity revisited. Arch Ophthalmol. 2005;123(7):991-999. doi:10.1001/archopht.123.7.991 16009843

[30] Early Treatment For Retinopathy Of Prematurity Cooperative Group. Revised indications for the treatment of retinopathy of prematurity: results of the early treatment for retinopathy of prematurity randomized trial. Arch Ophthalmol. 2003;121(12):1684-1694. doi:10.1001/archopht.121.12.1684 14662586

[31] Socialstyrelsen. Vård av extremt för tidigt födda barn. Accessed April 11, 2023. https://www.socialstyrelsen.se/globalassets/sharepoint-dokument/artikelkatalog/vagledning/2014-9-10.pdf#:~:text=Att%20f%C3%A5%20ett%20extremt%20f%C3%B6r%20tidigt%20f%C3%B6tt%20barn,och%20kontinuerligt%20informeras%20om%20barnets%20tillst%C3%A5nd%20och%20prognos

[32] Mihatsch WA, Braegger C, Bronsky J, . ESPGHAN/ESPEN/ESPR/CSPEN guidelines on pediatric parenteral nutrition. Clin Nutr. 2018;37(6, pt B):2303-2305. doi:10.1016/j.clnu.2018.05.029 30471662

[33] Holford TR. The analysis of rates and of survivorship using log-linear models. Biometrics. 1980;36(2):299-305. doi:10.2307/2529982 7407317

[34] Laird N, Olivier D. Covariance analysis of censored survival data using log-linear analysis techniques. J Am Stat Assoc. 1981;76(374):231-240. doi:10.1080/01621459.1981.10477634

[35] Whitehead J. Fitting Cox’s regression model to survival data using GLIM. J R Stat Soc [Ser A]. 1980;29(3):268-275. doi:10.2307/2346901

[36] Vanhaesebrouck S, Vanhole C, de Zegher F, Allegaert K. Influence of duration of parenteral nutrition on retinopathy of prematurity. Arch Dis Child Fetal Neonatal Ed. 2008;93(2):F170. doi:10.1136/adc.2007.128991 18296578

[37] Niwald A, Piotrowski A, Grałek M. Analysis of some of the possible neonatal risk factors of development of retinopathy of prematurity. Article in Polish. Klin Oczna. 2008;110(1-3):31-34. 18669080

[38] Petrachkova MS, Saidasheva EI, Petrachkov DV, Buyanovskaya SV. Modern approaches to predicting the development of active type 1 retinopathy of prematurity. Article in Russian. Vestn Oftalmol. 2019;135(4):50-59. doi:10.17116/oftalma201913504150 31573557

