# Supplementary material for: Prognostic Value of Parenteral Nutrition Duration on Risk of Retinopathy of Prematurity: Development and Validation of the Revised DIGIROP Clinical Decision Support Tool
Source: JAMA Ophthalmol. 2023 Jun 29;141(8):716–24. doi: 10.1001/jamaophthalmol.2023.2336 (PMC10311427; doi:10.1001/jamaophthalmol.2023.2336)
Supplement: Supplement 1. — eTable 1. Infant Characteristics for the Model Development Cohort and Temporal Validation Cohort by Parenteral Nutrition Duration eTable 2. Unadjusted and Adjusted Logistic Regression for Any Retinopathy of Prematurity (ROP) and ROP Treatment Studying Parenteral Nutrition as the Main Association Variable for the Model Development Cohort and Temporal Validation Cohort eTable 3. DIGIROP Pre-Screen 2.0 Prediction Model for Retinopathy of Prematurity Treatment Including Parenteral Nutrition Duration eTable 4. DIGIROP-Screen 2.0 Prediction Models for Retinopathy of Prematurity Treatment Including Parenteral Nutrition Duration eTable 5. Cutoffs for DIGIROP Pre-Screen 2.0 and DIGIROP Screen 2.0 Including Parenteral Nutrition Duration eTable 6. Sensitivity, Specificity, Cumulative Specificity, PPV, NPV, Model Accuracy, and 95% CI for DIGIROP Pre-Screen 2.0 and DIGIROP Screen 2.0 Including Parenteral Nutrition Duration for the Model Development and Temporal Validation Cohort eFigure 1. Bar Graphs for Percentage Infants With Any ROP by PN Duration, Gestational Age, and Overall eFigure 2. Bar Graphs for Percentage Infants with ROP Treatment by PN Duration, Gestational Age, and Sex eFigure 3. Estimated DIGIROP Pre-Screen 2.0 Probability for ROP Treatment Including Parenteral Nutrition Duration by Gestational Age eFigure 4. Calibration Plot for DIGIROP Pre-Screen 2.0 Including Parenteral Nutrition Duration for Model Development Cohort and Temporal Validation Cohort eFigure 5. Infants Discharged From Screening by PNA and GA eFigure 6. Internal Validation of DIGIROP Pre-Screen 2.0 and DIGIROP Screen 2.0 Including Parenteral Nutrition Duration Using 10-Fold Cross-Validation eFigure 7. Calibration Plot for DIGIROP-Screen 2.0 Including Parenteral Nutrition Duration for (A) Model Development Cohort and (B) Temporal Validation Cohort eFigure 8. Sensitivity and Specificity for DIGIROP Pre-Screen 2.0 and DIGIROP Screen 2.0 Including Parenteral Nutrition Duration [file jamaophthalmol-e232336-s001.pdf]

Supplemental Online Content

Pivodic A, Holmström G, Smith LEH, et al. Prognostic value of parenteral nutrition duration on risk of retinopathy of prematurity: development and validation of the revised DIGIROP clinical decision support tool. *JAMA Ophthalmol*. Published online June 29, 2023. doi:10.1001/jamaophthalmol.2023.2336

eTable 1. Infant Characteristics for the Model Development Cohort and Temporal Validation Cohort by Parenteral Nutrition Duration
eTable 2. Unadjusted and Adjusted Logistic Regression for Any Retinopathy of Prematurity (ROP) and ROP Treatment Studying Parenteral Nutrition as the Main Association Variable for the Model Development Cohort and Temporal Validation Cohort
eTable 3. DIGIROP Pre-Screen 2.0 Prediction Model for Retinopathy of Prematurity Treatment Including Parenteral Nutrition Duration
eTable 4. DIGIROP-Screen 2.0 Prediction Models for Retinopathy of Prematurity Treatment Including Parenteral Nutrition Duration
eTable 5. Cutoffs for DIGIROP Pre-Screen 2.0 and DIGIROP Screen 2.0 Including Parenteral Nutrition Duration
eTable 6. Sensitivity, Specificity, Cumulative Specificity, PPV, NPV, Model Accuracy, and 95% CI for DIGIROP Pre-Screen 2.0 and DIGIROP Screen 2.0 Including Parenteral Nutrition Duration for the Model Development and Temporal Validation Cohort
eFigure 1. Bar Graphs for Percentage Infants With Any ROP by PN Duration, Gestational Age, and Overall
eFigure 2. Bar Graphs for Percentage Infants with ROP Treatment by PN Duration, Gestational Age, and Sex
eFigure 3. Estimated DIGIROP Pre-Screen 2.0 Probability for ROP Treatment Including Parenteral Nutrition Duration by Gestational Age
eFigure 4. Calibration Plot for DIGIROP Pre-Screen 2.0 Including Parenteral Nutrition Duration for Model Development Cohort and Temporal Validation Cohort
eFigure 5. Infants Discharged From Screening by PNA and GA
eFigure 6. Internal Validation of DIGIROP Pre-Screen 2.0 and DIGIROP Screen 2.0 Including Parenteral Nutrition Duration Using 10-Fold Cross-Validation
eFigure 7. Calibration Plot for DIGIROP-Screen 2.0 Including Parenteral Nutrition Duration for (A) Model Development Cohort and (B) Temporal Validation Cohort
eFigure 8. Sensitivity and Specificity for DIGIROP Pre-Screen 2.0 and DIGIROP Screen 2.0 Including Parenteral Nutrition Duration

This supplementary material has been provided by the authors to give readers additional information about their work.

© 2023 Pivodic A et al. *JAMA Ophthalmology*.

eTable 1. Infant Characteristics for the Model Development Cohort and Temporal Validation Cohort by Parenteral Nutrition Duration

	Model Development Cohort				Temporal Validation Cohort			
Variable	PN <14d<br>N=5605	PN ≥14d<br>N=1640	PN ≥14d vs <14d<br>p-value	PN Unknown<br>N=1568	PN <14d<br>N=1623	PN ≥14d<br>N=668	PN ≥14d vs <14d<br>p-value	PN Unknown<br>N=34
Girls	2618 (46.7%)	729 (44.5%)	.11	661 (42.1%)	741 (45.7%)	309 (46.3%)	.79	13 (38.2%)
Gestational age (weeks)	29.3±2.0<br>29.6 (21.9 - 39.4)	26.2±2.1<br>26.0 (21.9 - 35.0)	<.001	28.7±2.3<br>29.1 (22.1 - 36.1)	28.8±1.8<br>29.1 (22.1 - 35.9)	26.1±2.1<br>25.9 (22.1 - 32.1)	<.001	28.3±2.4<br>29.1 (22.9 - 31.9)
Gestational age (weeks)			<.001				<.001	
<24	62 (1.1%)	227 (13.8%)		54 (3.4%)	18 (1.1%)	110 (16.5%)		3 (8.8%)
24-30	4508 (80.4%)	1380 (84.1%)		1207 (76.9%)	1521 (93.7%)	551 (82.5%)		29 (85.3%)
≥31	1035 (18.5%)	33 (2.0%)		308 (19.6%)	84 (5.2%)	7 (1.0%)		2 (5.9%)
Birth weight (g)	1287±358<br>1283 (382 - 3245)	851±292<br>797 (307 - 2320)	<.001	1209±387<br>1205 (410 - 2695)	1203±307<br>1203 (450 - 2405)	835±328<br>760 (340 - 3540)	<.001	1145±375<br>1150 (370 - 1800)
Birth weight SDS<br>(*N=10665*)	-1.07±1.40<br>-0.8 (-8.1 - 4.9)	-1.21±1.57<br>-0.8 (-9.1 - 3.4)	.05	-1.12±1.42<br>-0.8 (-8.7 - 4.0)	-1.12±1.40<br>-0.8 (-6.9 - 3.4)	-1.29±1.70<br>-0.9 (-6.7 - 5.4)	.23	-1.20±1.84<br>-0.6 (-5.5 - 1.4)
Any ROP	999 (17.8%)	1063 (64.8%)	<.001	354 (22.6%)	341 (21.0%)	414 (62.0%)	<.001	8 (23.5%)
Maximum ROP stage			<.001				<.001	
No ROP	4606 (82.2%)	577 (35.2%)		1215 (77.4%)	1282 (79.0%)	254 (38.0%)		26 (76.5%)
Max stage 1	399 (7.1%)	210 (12.8%)		106 (6.8%)	160 (9.9%)	80 (12.0%)		1 (2.9%)
Max stage 2 not treated	385 (6.9%)	361 (22.0%)		128 (8.2%)	107 (6.6%)	118 (17.7%)		3 (8.8%)
Max stage 3 not treated	123 (2.2%)	198 (12.1%)		58 (3.7%)	51 (3.1%)	91 (13.6%)		0 (0.0%)
Max stage 5 not treated	0 (0.0%)	1 (0.1%)		0 (0.0%)	0 (0.0%)	0 (0.0%)		0 (0.0%)
Treated ROP	92 (1.6%)	293 (17.9%)		62 (4.0%)	23 (1.4%)	125 (18.7%)		4 (11.8%)
PNA weeks at first ROP diagnosis<br>(*N=3179*)	7.9±2.2<br>7.6 (1.3 - 17.6)	9.1±2.2<br>9.0 (4.0 - 24.7)	<.001	8.6±2.4<br>8.3 (4.1 - 17.3)	7.8±2.3<br>7.6 (0.9 - 16.6)	9.3±2.1<br>9.1 (4.9 - 17.6)	<.001	8.9±1.3<br>8.9 (6.6 - 10.6)
PNA weeks at first ROP treatment<br>(*N=599*)	13.4±3.0<br>12.9 (6.3 - 20.7)	12.7±2.8<br>12.4 (7.0 - 28.3)	.04	12.9±3.0<br>12.4 (7.9 - 19.9)	13.6±3.5<br>12.6 (9.4 - 24.3)	13.2±3.3<br>12.6 (7.7 - 26.3)	.62	12.7±1.4<br>13.2 (10.7 - 13.9)
Weeks between first ROP diagnosis and treatment (*N=599*)	4.8±3.0<br>4.4 (0.1 - 13.9)	3.6±2.7<br>3.0 (0.1 - 18.9)	<.001	3.8±2.9<br>3.2 (0.1 - 11.6)	5.0±3.5<br>4.1 (0.3 - 15.9)	3.6±3.3<br>3.0 (0.0 - 19.1)	.03	3.6±1.1<br>3.8 (2.4 - 4.6)
Days on parenteral nutrition<br>(*N=9536*)	4.0±4.1<br>3 (0 - 13)	30.3±22.7<br>23 (14 - 279)	<.001	NA	6.0±3.9<br>6 (0 - 13)	32.2±26.6<br>23 (14 - 257)	<.001	NA

PN = Parenteral nutrition; d = days; SDS = Standard deviation score; ROP = Retinopathy of prematurity

Data are presented as mean±standard deviation, median (range), or number (percentage). For test between two groups with respect to dichotomous variables Fisher's exact test was used, for ordered categorical variables Mantel-Haenszel Chi-square trend test, and for continuous variables Mann-Whitney U-test.

© 2023 Pivodic A et al. *JAMA Ophthalmology*.

eTable 2. Unadjusted and Adjusted Logistic Regression for Any Retinopathy of Prematurity (ROP) and ROP Treatment Studying Parenteral Nutrition as the Main Association Variable for the Model Development Cohort and Temporal Validation Cohort

				Unadjusted			Adjusted for GA, BW and Sex		
**Outcome Variable**	**Main Association Variable**	**Values**	**N (%) Events**	**OR (95% CI)**	***P*-value**	**AUC**	**aOR (95% CI)**	***P*-value**	**AUC**
**Model Development Cohort**									
Any ROP	Days on parenteral nutrition (per 7 days increase)			1.80 (1.73 - 1.88)	<.001	0.77	1.18 (1.14 - 1.23)	<.001	0.88
	Days on parenteral nutrition (categorized)	<14	999 (17.8%)	Reference			Reference		
		≥14	1063 (64.8%)	8.49 (7.52 - 9.60)	<.001	0.69	1.96 (1.68 - 2.28)	<.001	0.87
		Unknown	354 (22.6%)	1.34 (1.17 - 1.54)	<.001		0.87 (0.73 - 1.03)	.10	
ROP treatment	Days on parenteral nutrition (per 7 days increase)			1.41 (1.36 - 1.46)	<.001	0.83	1.15 (1.11 - 1.19)	<.001	0.93
	Days on parenteral nutrition (categorized)	<14	92 (1.6%)	Reference			Reference		
		≥14	293 (17.9%)	13.04 (10.24 - 16.60)	<.001	0.77	2.14 (1.62 - 2.81)	<.001	0.93
		Unknown	62 (4.0%)	2.47 (1.78 - 3.42)	<.001		1.28 (0.89 - 1.83)	.19	
**Temporal Validation Cohort**									
Any ROP	Days on parenteral nutrition (per 7 days increase)			1.58 (1.49 - 1.68)	<.001	0.76	1.13 (1.08 - 1.19)	<.001	0.84
	Days on parenteral nutrition (categorized)	<14	341 (21.0%)	Reference			Reference		
		≥14	414 (62.0%)	6.13 (5.03 - 7.46)	<.001	0.69	1.63 (1.27 - 2.10)	<.001	0.84
		Unknown	8 (23.5%)	1.16 (0.52 - 2.58)	.72		0.71 (0.27 - 1.91)	.50	
ROP treatment	Days on parenteral nutrition (per 7 days increase)			1.25 (1.20 - 1.31)	<.001	0.85	1.07 (1.02 - 1.13)	.01	0.93
	Days on parenteral nutrition (categorized)	<14	23 (1.4%)	Reference			Reference		
		≥14	125 (18.7%)	16.01 (10.16 - 25.24)	<.001	0.79	2.49 (1.49 - 4.17)	<.001	0.93
		Unknown	4 (11.8%)	9.27 (3.02 - 28.47)	<.001		5.09 (1.21 - 21.35)	.03	

ROP = Retinopathy of prematurity; PN = Parenteral nutrition; GA = Gestational age; BW = Birth weight; OR = Odds ratio; aOR = Adjusted odds ratio; AUC = Area under receiver operating characteristic curve

© 2023 Pivodic A et al. *JAMA Ophthalmology*.

eTable 3. DIGIROP Pre-Screen 2.0 Prediction Model for Retinopathy of Prematurity Treatment Including Parenteral Nutrition Duration

Predictor	Estimate	SE	HR (95% CI)	*P*-value
Intercept	-17.2284	2.8183		<.001
PNA birth to 8w (per 1w inc.)	1.6218	0.3523	5.06 (2.54 - 10.10)	<.001
PNA 8 to 12w (per 1w inc.)	0.4897	0.0483	1.63 (1.48 - 1.79)	<.001
PNA 12w and onwards (per 1w inc.)	-0.3505	0.0234	0.70 (0.67 - 0.74)	<.001
GA below 27w (per 1w inc.)	0.0446	0.1245	1.05 (0.82 - 1.33)	.72
GA above 27w (per 1w inc.)	1.8942	0.5803	6.65 (2.13 - 20.73)	.001
Sex (0=boy, 1=girl)	-0.9740	0.2767	0.38 (0.22 - 0.65)	<.001
BW (per 100g inc.)	-0.3331	0.0538	0.72 (0.65 - 0.80)	<.001
PN ≥14d vs <14d	0.4744	0.1509	1.61 (1.20 - 2.16)	.002
PN Unknown vs <14d	0.1963	0.1662	1.22 (0.88 - 1.69)	.24
Interaction: PNA (per 1w inc.) * GA above 27w (per 1w inc.)	-0.1579	0.0505	0.85 (0.77 - 0.94)	.002
Interaction: Sex (0=boy, 1=girl) * PN ≥14d vs <14d	0.4317	0.2120	1.54 (1.02 - 2.33)	.04
Interaction: BW (per 100g inc.) * GA (per 1w inc.)	-0.0709	0.0184	0.93 (0.90 - 0.97)	<.001
Interaction: Sex (0=boy, 1=girl) * GA below 27w (per 1w inc.)	-0.1528	0.0740	0.86 (0.74 - 0.99)	.04

SE = Standard error; HR = Hazard ratio; PNA = Parenteral nutrition; GA = Gestational age; BW = Birth weight; PN = Parenteral nutrition; d = days; w = weeks; inc. = increase.

[1] Centered at 28 weeks GA

Area under the receiver operating characteristic curve (AUC) is 0.93.

Hosmer-Lemeshow test on the logodds of estimated probability resulted in p=0.61.

© 2023 Pivodic A et al. *JAMA Ophthalmology*.

eTable 4. DIGIROP Screen 2.0 Prediction Models for Retinopathy of Prematurity Treatment Including Parenteral Nutrition Duration

Model for:	Variables	Estimate (SE)	p-value	Hosmer-Lemeshow Goodness-of-fit test	AUC
**PNA 6 weeks**	Intercept	0.0898 (0.0892)	0.31	0.87	0.93
	logodds(DIGIROP Pre-Screen 2.0)	1.0226 (0.0443)	<.001		
	FirstROP	0.7760 (0.3016)	.01		
**PNA 7 weeks**	Intercept	0.0801 (0.0902)	.37	0.67	0.93
	logodds(DIGIROP Pre-Screen 2.0)	1.0414 (0.0454)	<.001		
	FirstROP	0.5992 (0.1787)	<.001		
**PNA 8 weeks**	Intercept	0.1399 (0.1038)	.18	0.71	0.94
	logodds(DIGIROP Pre-Screen 2.0)	1.2094 (0.0610)	<.001		
	FirstROP	-0.4783 (0.9830)	.63		
	FirstROP x FirstROPWeek	0.0589 (0.1357)	.66		
	logodds(DIGIROP Pre-Screen 2.0) x FirstROP x FirstROPWeek	-0.0735 (0.0145)	<.001		
**PNA 9 weeks**	Intercept	0.0276 (0.1180)	.82	0.95	0.94
	logodds(DIGIROP Pre-Screen 2.0)	1.2662 (0.0717)	<.001		
	FirstROP	0.8544 (0.6936)	.22		
	FirstROP x FirstROPWeek	-0.0889 (0.0878)	.31		
	logodds(DIGIROP Pre-Screen 2.0) x FirstROP x FirstROPWeek	-0.0604 (0.0136)	<.001		
**PNA 10 weeks**	Intercept	-0.3705 (0.1502)	.01	1.00	0.95
	logodds(DIGIROP Pre-Screen 2.0)	1.3305 (0.0956)	<.001		
	FirstROP	0.7799 (0.5406)	.15		
	FirstROP x FirstROPWeek	-0.0097 (0.0642)	.88		
	logodds(DIGIROP Pre-Screen 2.0) x FirstROP x FirstROPWeek	-0.0548 (0.0141)	<.001		
**PNA 11 weeks**	Intercept	-1.0938 (0.2141)	<.001	0.81	0.95
	logodds(DIGIROP Pre-Screen 2.0)	1.2653 (0.1236)	<.001		
	FirstROP	1.3043 (0.4991)	.009		
	FirstROP x FirstROPWeek	-0.0092 (0.0563)	.87		
	logodds(DIGIROP Pre-Screen 2.0) x FirstROP x FirstROPWeek	-0.0434 (0.0161)	.007		
**PNA 12 weeks**	Intercept	-1.6328 (0.3047)	<.001	0.92	0.95
	logodds(DIGIROP Pre-Screen 2.0)	1.2201 (0.1537)	<.001		
	FirstROP	1.8943 (0.5005)	<.001		
	FirstROP x FirstROPWeek	-0.0589 (0.0550)	.28		
	logodds(DIGIROP Pre-Screen 2.0) x FirstROP x FirstROPWeek	-0.0436 (0.0184)	.02		
**PNA 13 weeks**	Intercept	-2.0790 (0.4128)	<.001	0.97	0.94
	logodds(DIGIROP Pre-Screen 2.0)	1.2852 (0.2038)	<.001		
	FirstROP	1.8992 (0.5684)	<.001		
	FirstROP x FirstROPWeek	-0.0753 (0.0611)	.22		
	logodds(DIGIROP Pre-Screen 2.0) x FirstROP x FirstROPWeek	-0.0640 (0.0229)	.005		
**PNA 14 weeks**	Intercept	-2.5952 (0.5134)	<.001	1.00	0.94

© 2023 Pivodic A et al. *JAMA Ophthalmology*.

Model for:	Variables	Estimate (SE)	p-value	Hosmer-Lemeshow Goodness-of-fit test	AUC
	logodds(DIGIROP Pre-Screen 2.0)	1.1839 (0.2224)	<.001		
	FirstROP	2.1799 (0.6152)	<.001		
	FirstROP x FirstROPWeek	-0.0826 (0.0643)	.20		
	logodds(DIGIROP Pre-Screen 2.0) x FirstROP x FirstROPWeek	-0.0562 (0.0246)	.02		
ROP = Retinopathy of prematurity; PNA = Postnatal age; logodds(DIGIROP Pre-Screen 2.0) = log([DIGIROP Pre-Screen 2.0 risk estimate]/(1-[DIGIROP Pre-Screen 2.0 risk estimate])); FirstROP = First ROP diagnosed no(0)/yes(1); FirstROPWeek = Postnatal week when first ROP is diagnosed					

© 2023 Pivodic A et al. *JAMA Ophthalmology*.

eTable 5. Cutoffs for DIGIROP Pre-Screen 2.0 and DIGIROP Screen 2.0 Including Parenteral Nutrition Duration

GA at birth	Birth	PNA 6 weeks	PNA 7 weeks	PNA 8 weeks	PNA 9 weeks	PNA 10 weeks	PNA 11 weeks	PNA 12 weeks	PNA 13 weeks	PNA 14 weeks
**21**	0.05	0.05	0.05	0.05	0.05	0.05	0.05	0.05	0.05	0.05
**22**	0.05	0.05	0.05	0.05	0.05	0.05	0.05	0.0407	0.0211	0.05
**23**	0.05	0.05	0.05	0.05	0.05	0.05	0.05	0.0323	0.0164	0.0094
**24**	0.05	0.05	0.05	0.05	0.05	0.05	0.046	0.0257	0.0218	0.012
**25**	0.0374	0.0318	0.0294	0.0169	0.0119	0.0203	0.0104	0.0068	0.0312	0.0249
**26**	0.0172	0.0135	0.0123	0.0108	0.0074	0.006	0.0031	0.0018	0.0007	0.0005
**27**	0.005	0.0034	0.003	0.0011	0.0006	0.0002	0.0073	0.0059	0.0133	0.0117
**28**	0.0035	0.0023	0.002	0.0007	0.0031	0.0013	0.0007	0.0004	0.006	0.0053
**29**	0.0017	0.0015	0.0013	0.0018	0.0016	0.0035	0.0025	0.0023	0.002	0.0019
**31**	0.0003	0.0003	0.0003	0.0003	0.0003	0.0003	0.0003	0.0003	0.0003	0.0003

© 2023 Pivodic A et al. *JAMA Ophthalmology*.

eTable 6. Sensitivity, Specificity, Cumulative Specificity, PPV, NPV, Model Accuracy, and 95% CI for DIGIROP Pre-Screen 2.0 and DIGIROP Screen 2.0 Including Parenteral Nutrition Duration for the Model Development and Temporal Validation Cohort

	Sensitivity		Specificity	Cumulative Specificity	Positive Predictive Value	Negative Predictive Value	Model Accuracy	Area under the ROC Curve
Cohort, Model and Timepoint	n/N	% (95% CI)	% (95% CI)	% (95% CI)	% (95% CI)	% (95% CI)	% (95% CI)	AUC (95% CI)
**Model Development Cohort**								
**DIGIROP Pre-Screen 2.0**	447/447	100.0 (99.2-100.0)	48.5 (47.4-49.5)	48.5 (47.4-49.5)	9.4 (8.6-10.3)	100.0 (99.9-100.0)	51.1 (50.0-52.1)	0.93 (0.92 - 0.94)
**DIGIROP Screen 2.0 PNA6w**	447/447	100.0 (99.2-100.0)	44.9 (43.8-46.0)	48.6 (47.5-49.7)	8.8 (8.1-9.7)	100.0 (99.9-100.0)	47.7 (46.6-48.7)	0.93 (0.92 - 0.94)
**DIGIROP Screen 2.0 PNA7w**	446/446	100.0 (99.2-100.0)	45.0 (43.9-46.1)	48.9 (47.8-50.0)	8.8 (8.1-9.7)	100.0 (99.9-100.0)	47.8 (46.7-48.8)	0.93 (0.92 - 0.94)
**DIGIROP Screen 2.0 PNA8w**	440/440	100.0 (99.2-100.0)	51.3 (50.2-52.3)	55.6 (54.5-56.7)	9.7 (8.9-10.6)	100.0 (99.9-100.0)	53.7 (52.7-54.8)	0.94 (0.93 - 0.95)
**DIGIROP Screen 2.0 PNA9w**	423/423	100.0 (99.1-100.0)	59.5 (58.4-60.5)	61.6 (60.5-62.6)	11.1 (10.1-12.1)	100.0 (99.9-100.0)	61.4 (60.4-62.5)	0.94 (0.93 - 0.95)
**DIGIROP Screen 2.0 PNA10w**	405/405	100.0 (99.1-100.0)	62.6 (61.6-63.7)	64.5 (63.5-65.5)	11.5 (10.4-12.6)	100.0 (99.9-100.0)	64.3 (63.3-65.3)	0.95 (0.94 - 0.95)
**DIGIROP Screen 2.0 PNA11w**	339/339	100.0 (98.9-100.0)	69.3 (68.3-70.3)	71.8 (70.8-72.8)	11.6 (10.5-12.9)	100.0 (99.9-100.0)	70.5 (69.5-71.4)	0.95 (0.94 - 0.96)
**DIGIROP Screen 2.0 PNA12w**	261/261	100.0 (98.6-100.0)	67.9 (66.9-68.9)	72.1 (71.1-73.1)	8.9 (7.9-9.9)	100.0 (99.9-100.0)	68.9 (67.9-69.9)	0.95 (0.94 - 0.96)
**DIGIROP Screen 2.0 PNA13w**	174/174	100.0 (97.9-100.0)	75.9 (75.0-76.9)	77.9 (77.0-78.8)	8.0 (6.9-9.2)	100.0 (99.9-100.0)	76.4 (75.5-77.3)	0.94 (0.93 - 0.95)
**DIGIROP Screen 2.0 PNA14w**	137/137	100.0 (97.3-100.0)	75.2 (74.3-76.1)	78.0 (77.1-78.9)	6.2 (5.2-7.3)	100.0 (99.9-100.0)	75.6 (74.7-76.5)	0.94 (0.93 - 0.95)
**Temporal Validation Cohort**								
**DIGIROP Pre-Screen 2.0**	152/152	100.0 (97.6-100.0)	39.4 (37.3-41.5)	39.4 (37.3-41.5)	10.3 (8.8-12.0)	100.0 (99.6-100.0)	43.4 (41.3-45.4)	0.93 (0.91 - 0.94)
**DIGIROP Screen 2.0 PNA6w**	152/152	100.0 (97.6-100.0)	35.1 (33.1-37.1)	39.5 (37.5-41.6)	9.7 (8.3-11.3)	100.0 (99.5-100.0)	39.3 (37.3-41.3)	0.93 (0.91 - 0.94)
**DIGIROP Screen 2.0 PNA7w**	152/152	100.0 (97.6-100.0)	34.9 (32.9-36.9)	39.7 (37.6-41.8)	9.7 (8.3-11.3)	100.0 (99.5-100.0)	39.1 (37.1-41.2)	0.93 (0.92 - 0.95)
**DIGIROP Screen 2.0 PNA8w**	151/151	100.0 (97.6-100.0)	41.7 (39.7-43.8)	46.8 (44.7-49.0)	10.7 (9.1-12.4)	100.0 (99.6-100.0)	45.5 (43.5-47.6)	0.93 (0.92 - 0.95)
**DIGIROP Screen 2.0 PNA9w**	148/148	100.0 (97.5-100.0)	49.4 (47.3-51.5)	52.0 (49.8-54.1)	11.9 (10.1-13.8)	100.0 (99.7-100.0)	52.6 (50.6-54.7)	0.94 (0.93 - 0.95)
**DIGIROP Screen 2.0 PNA10w**	137/137	100.0 (97.3-100.0)	53.0 (50.9-55.1)	55.6 (53.5-57.7)	11.8 (10.0-13.8)	100.0 (99.7-100.0)	55.8 (53.7-57.8)	0.94 (0.92 - 0.95)
**DIGIROP Screen 2.0 PNA11w**	118/118	100.0 (96.9-100.0)	60.6 (58.5-62.6)	64.1 (62.0-66.1)	12.1 (10.1-14.3)	100.0 (99.7-100.0)	62.6 (60.6-64.6)	0.94 (0.92 - 0.95)
**DIGIROP Screen 2.0 PNA12w**	86/86	100.0 (95.8-100.0)	59.3 (57.2-61.4)	64.5 (62.5-66.5)	8.9 (7.2-10.8)	100.0 (99.7-100.0)	60.9 (58.8-62.9)	0.94 (0.93 - 0.95)
**DIGIROP Screen 2.0 PNA13w**	70/70	100.0 (94.9-100.0)	69.8 (67.8-71.7)	72.2 (70.3-74.1)	9.6 (7.6-12.0)	100.0 (99.8-100.0)	70.8 (68.8-72.6)	0.94 (0.93 - 0.96)
**DIGIROP Screen 2.0 PNA14w**	44/44	100.0 (92.0-100.0)	69.2 (67.2-71.1)	72.4 (70.5-74.3)	6.2 (4.5-8.2)	100.0 (99.8-100.0)	69.8 (67.8-71.7)	0.96 (0.94 - 0.97)

ROC = Receiver operating characteristic; AUC = Area under the ROC curve

© 2023 Pivodic A et al. *JAMA Ophthalmology*.

eFigure 1. Bar Graphs for Percentage Infants With Any ROP by PN Duration, Gestational Age, and Overall

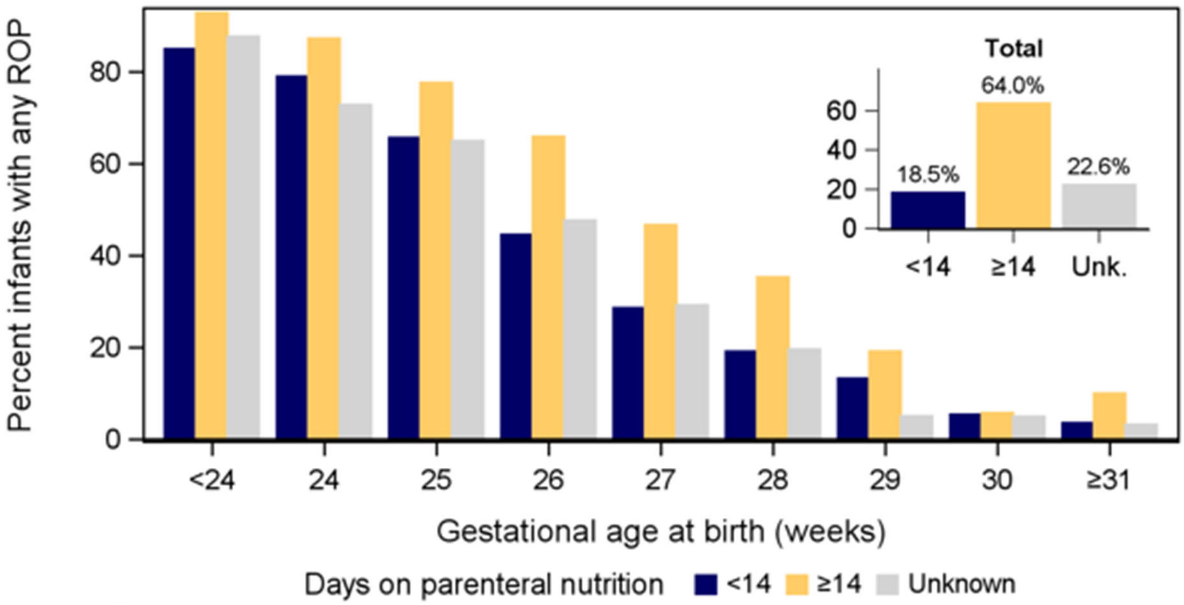

	<24	24	25	26	27	28	29	30	≥31
<14	68 (85.0%)	113 (79.0%)	178 (65.7%)	226 (44.6%)	210 (28.6%)	209 (19.1%)	200 (13.3%)	96 (5.4%)	40 (3.6%)
≥14	313 (92.9%)	316 (87.3%)	330 (77.6%)	269 (65.9%)	141 (46.7%)	71 (35.3%)	28 (19.2%)	5 (5.7%)	4 (10.0%)
Unknown	50 (87.7%)	51 (72.9%)	65 (65.0%)	62 (47.7%)	57 (29.2%)	40 (19.6%)	13 (5.1%)	14 (5.0%)	10 (3.2%)

eFigure 2. Bar Graphs for Percentage Infants with ROP Treatment by PN Duration, Gestational Age, and Sex

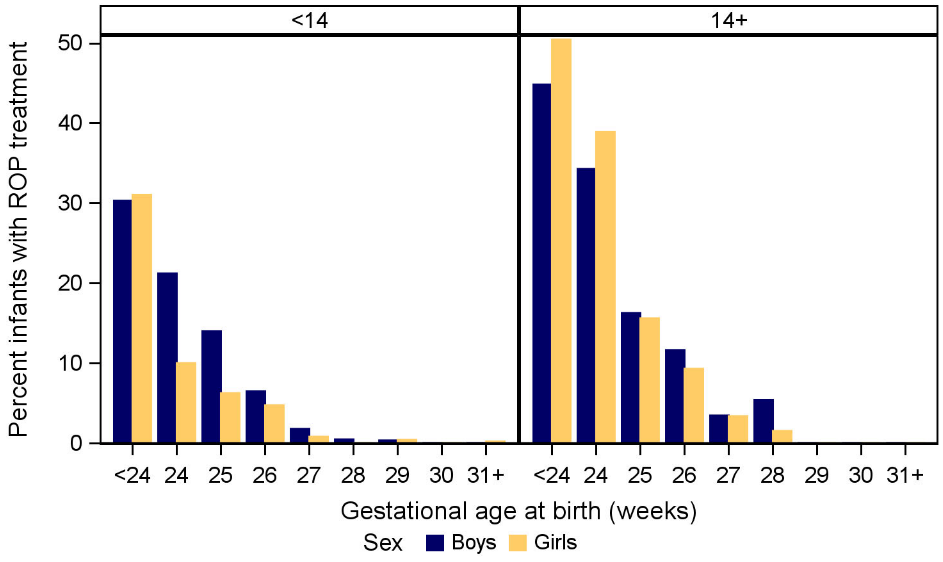

© 2023 Pivodic A et al. *JAMA Ophthalmology*.

eFigure 3. Estimated DIGIROP Pre-Screen 2.0 Probability for ROP Treatment Including Parenteral Nutrition Duration by Gestational Age

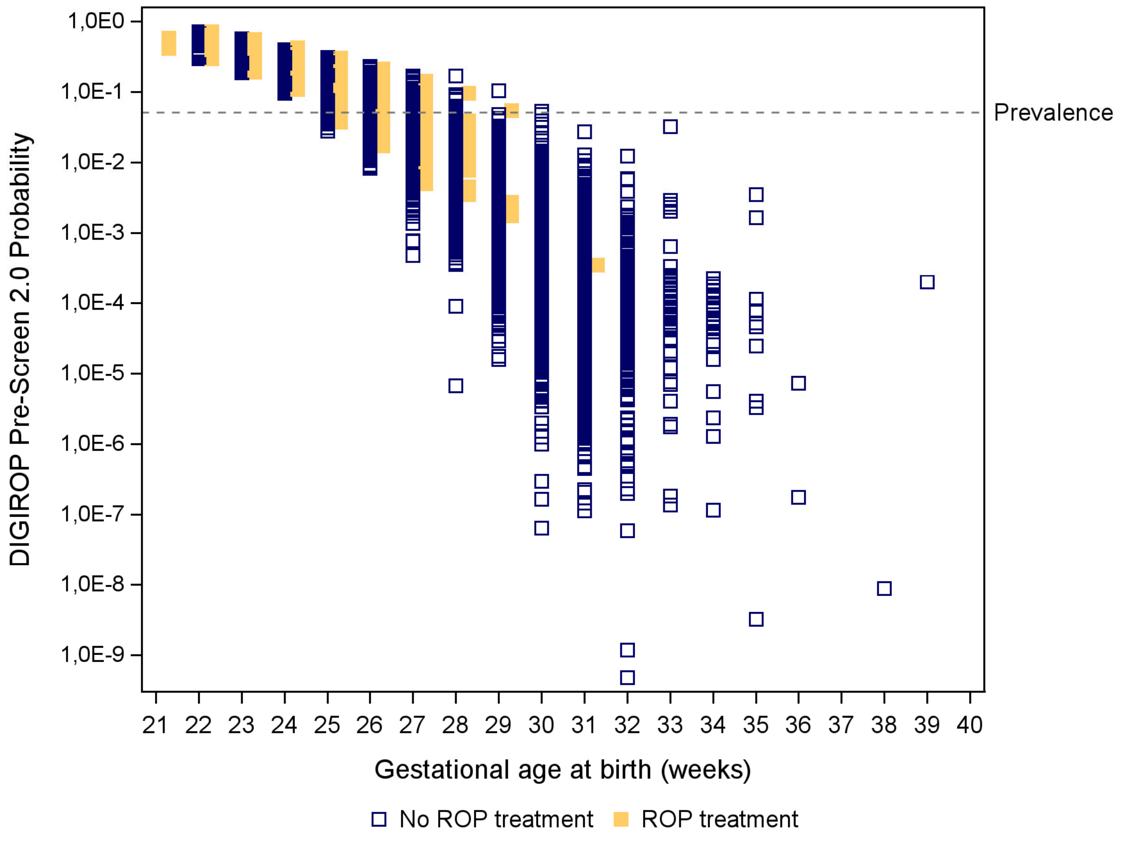

© 2023 Pivodic A et al. *JAMA Ophthalmology*.

eFigure 4. Calibration Plot for DIGIROP Pre-Screen 2.0 Including Parenteral Nutrition Duration for Model Development Cohort and Temporal Validation Cohort

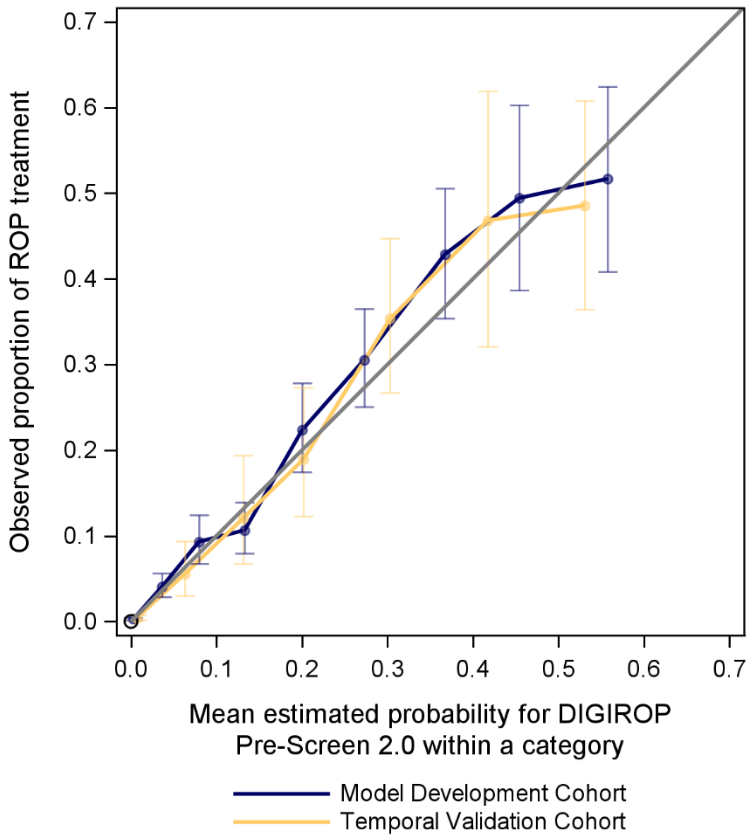

© 2023 Pivodic A et al. *JAMA Ophthalmology*.

eFigure 5. Infants Discharged From Screening by PNA and GA

PNA = Postnatal age; GA = Gestational age; ROP = Retinopathy of prematurity

A) Model Development Cohort at Pre-Screen by GA

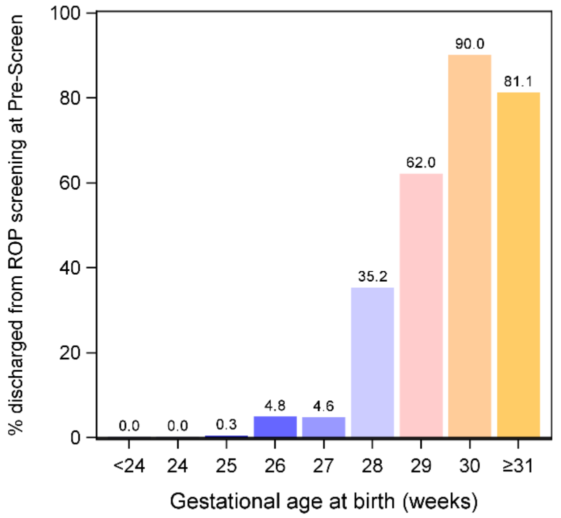

B) Temporal Validation Cohort at Pre-Screen by GA

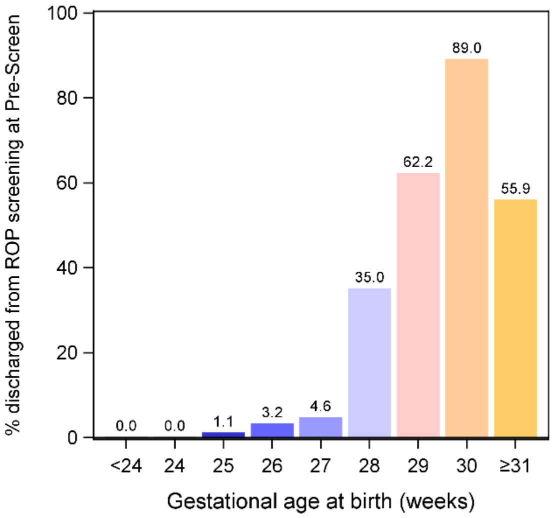

C) Model Development Cohort by PNA and GA

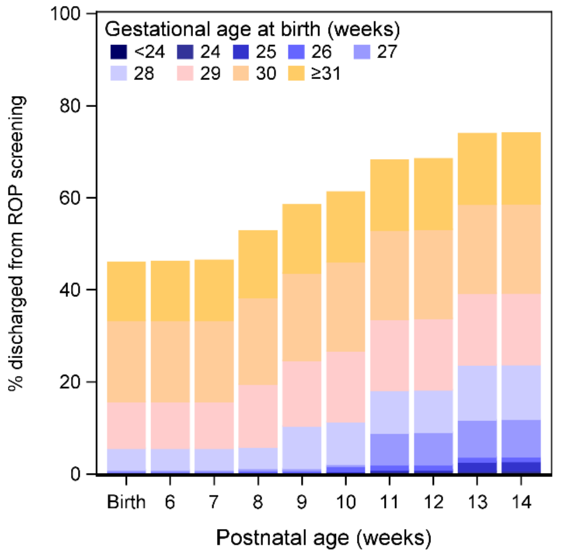

D) Temporal Validation Cohort by PNA and GA

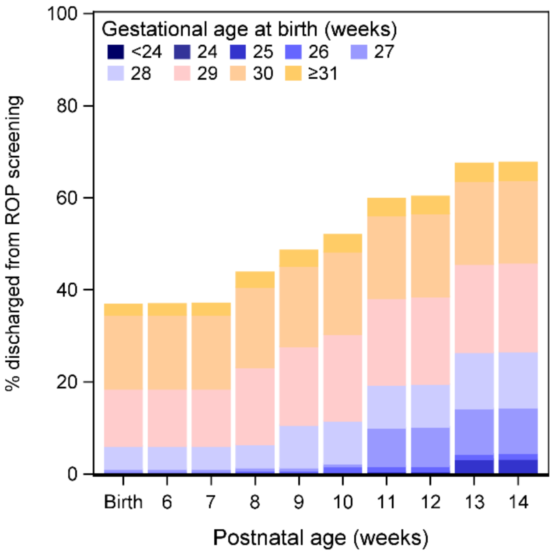

© 2023 Pivodic A et al. *JAMA Ophthalmology*.

eFigure 6. Internal Validation of DIGIROP Pre-Screen 2.0 and DIGIROP Screen 2.0 Including Parenteral Nutrition Duration Using 10-Fold Cross-Validation

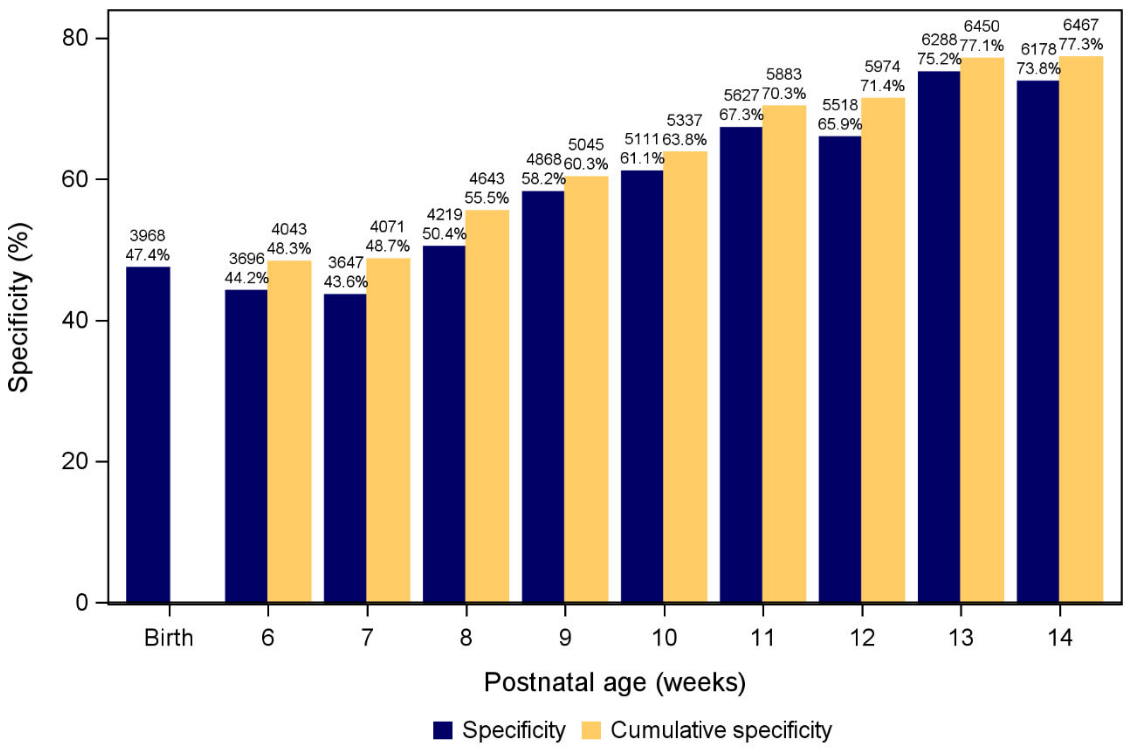

© 2023 Pivodic A et al. *JAMA Ophthalmology*.

eFigure 7. Calibration Plot for DIGIROP Screen 2.0 Including Parenteral Nutrition Duration for (A) Model Development Cohort and (B) Temporal Validation Cohort

A)

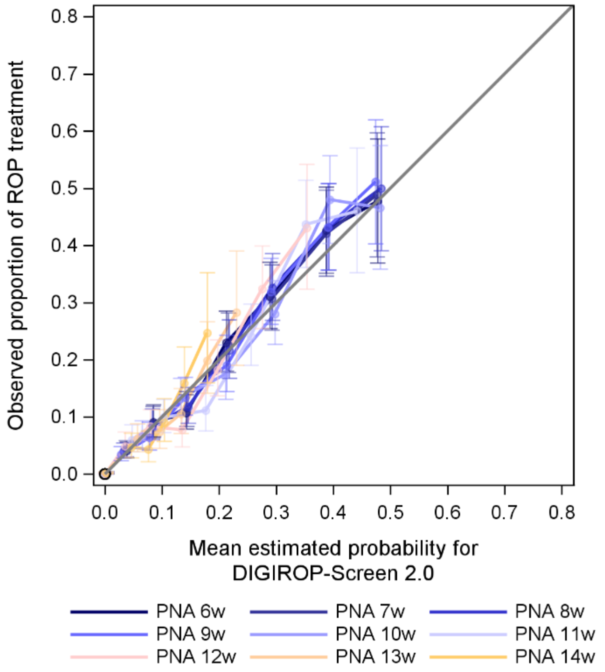

B)

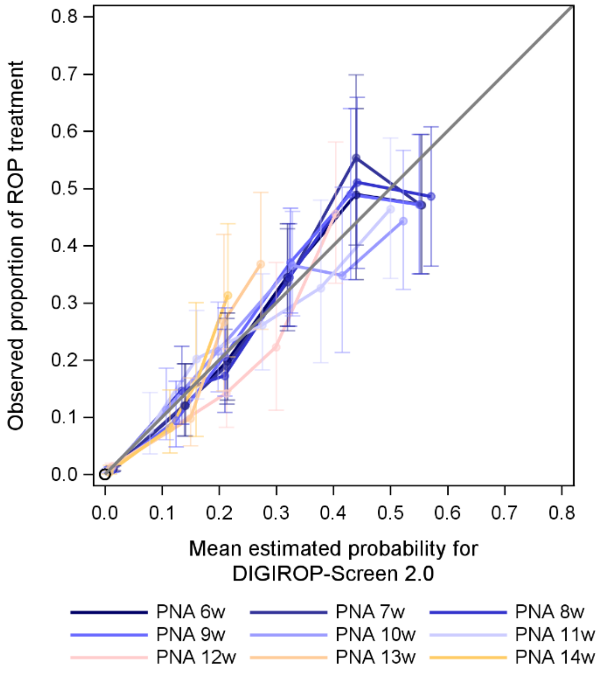

© 2023 Pivodic A et al. *JAMA Ophthalmology*.

eFigure 8. Sensitivity and Specificity for DIGIROP Pre-Screen 2.0 and DIGIROP Screen 2.0 Including Parental Nutrition Duration

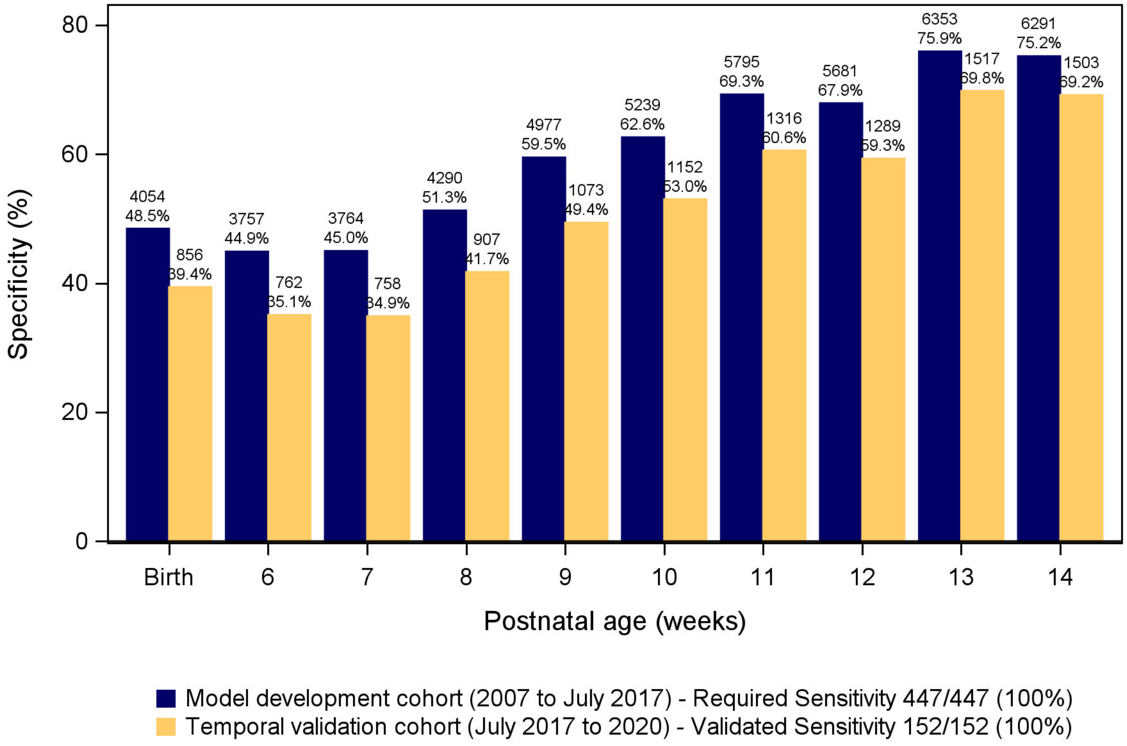

© 2023 Pivodic A et al. *JAMA Ophthalmology*.
