# Supplementary material for: Prognostic Value of Parenteral Nutrition Duration on Risk of Retinopathy of Prematurity: Development and Validation of the Revised DIGIROP Clinical Decision Support Tool
Source: JAMA Ophthalmol. 2023 Jun 29;141(8):716–24. doi: 10.1001/jamaophthalmol.2023.2336 (PMC10311427; doi:10.1001/jamaophthalmol.2023.2336)
Supplement: Supplement 2. — Data Sharing Statement [file jamaophthalmol-e232336-s002.pdf]

Data Sharing Statement

Pivodic. Prognostic Value of Parenteral Nutrition Duration on Risk of Retinopathy of Prematurity. *JAMA Ophthalmol*. Published June 29, 2023.
doi:10.1001/jamaophthalmol.2023.2336

Data
Data available: No
